# Autoantibodies in chronic non-infectious respiratory diseases: a scoping review

**DOI:** 10.3389/fimmu.2026.1809141

**Published:** 2026-07-28

**Authors:** Ke Li Chow, Matthijs Bekkers, Theo de Malmanche, Kathryn Patchett, Peter A. B. Wark, Gerard E. Kaiko

**Affiliations:** 1 School of Biomedical Sciences and Pharmacy, University of Newcastle, Newcastle, NSW, Australia; 2 Immune Health Program, Hunter Medical Research Institute, Newcastle, NSW, Australia; 3 Department of Immunology, John Hunter Hospital, Newcastle, NSW, Australia; 4 School of Medicine and Public Health, University of Newcastle, Newcastle, NSW, Australia; 5 Department of Immunology, New South Wales Health Pathology, Newcastle, NSW, Australia; 6 Department of Respiratory and Sleep Medicine, Alfred Health, Melbourne, VIC, Australia

**Keywords:** asthma, autoantibodies, autoantigens, autoimmune, bronchiectasis, COPD, cystic fibrosis, respiratory diseases

## Abstract

We performed a scoping review assessing autoantibodies across chronic non-infectious respiratory diseases including idiopathic pulmonary fibrosis, idiopathic interstitial pneumonias, chronic obstructive pulmonary disease, cystic fibrosis, asthma, bronchiectasis, sarcoidosis, and silicosis. Databases were searched for case-control and cohort studies investigating autoantibodies in the context of these respiratory diseases from inception to 13 September 2024. Of 5157 records identified, 79 studies were included, including three studies identified through citation review. Overall, few studies employed systematic, unbiased investigation of autoantibodies with most taking a predefined targeted approach. There was limited evidence in the literature of studies that also included functional validation of their findings. Despite statistical correlations between specific autoantibodies and certain respiratory diseases compared to healthy controls, the clinical relevance of many novel autoantibodies remains uncertain beyond a small number already utilized in standard clinical practice. This highlights a major gap in current understanding and priority areas for future research for endotyping by autoantibodies. This review summarises the current state of evidence on autoantibodies across chronic non-infectious respiratory diseases.

## Introduction

Autoantibodies are defined as antibodies produced against self-antigens due to loss of tolerance. The use of autoantibodies as biomarkers in diagnostic clinical practice to characterise and subclassify systemic autoimmune rheumatic diseases is well established. In some contexts, these autoantibodies are simply bystanders and do not exert meaningful functional responses, but are biomarkers of disease.

However, in some situations, autoantibodies can be pathogenic and act as functional mediators of tissue damage, as is the case for anti-GBM disease wherein animal models have demonstrated that the autoantibodies are pathogenic and standard clinical practice involves treatment that blocks or depletes the autoantibodies to reduce this tissue damage (1). In other cases, pathogenic autoantibodies may result in secondary or ‘acquired’ immunodeficiency where blockade of immune mediators leads to greater susceptibility to infection and then subsequent tissue damage. In the case of the latter increasingly autoantibodies to cytokines and complement factors have been identified (2). For example, autoantibodies to type I interferons (IFNs) confer an increased risk of severe COVID-19 (3) by suppressing the innate antiviral response and these recent scientific breakthroughs have stimulated further research efforts in this area. As a result, similar mechanisms have been identified in other conditions such as tick-borne encephalitis (4), West Nile virus encephalitis (5) and reactions to yellow fever vaccine (6). The expanding list of acquired immunodeficiencies due to autoantibodies, termed phenocopies of inborn errors of immunity (IEI) (7), together with the prevalence of respiratory disease comorbidities in systemic autoimmune conditions, raises the possibility of an under-recognised autoantibody-driven pathogenesis in chronic respiratory conditions. Furthermore, the presence of autoantibodies in chronic respiratory diseases may provide important clinical information even when they may not be driving disease pathogenesis. These autoantibodies may represent downstream effects of a specific disease process in heterogenous respiratory diseases and thus act as potential biomarkers that can be used for disease endotyping for prognosis or treatment prediction. For all of these reasons autoantibodies in chronic respiratory diseases represent an area of burgeoning clinical interest. This scoping review was conducted to systematically map the existing literature assessing autoantibodies across respiratory diseases and identify existing knowledge gaps.

## Methods

The study was drafted and reported according to the Preferred Reporting Items for Systematic Reviews and Meta-Analyses - Extension for Scoping Reviews (PRISMA-ScR) (8). To be included in the review, studies needed to focus on autoantibodies in respiratory diseases as the primary theme. During the initial search and screening process, the review was designed with a broad scope to encompass both infectious and non-infectious respiratory diseases. Accordingly, studies of interest included those investigating COVID-19, idiopathic pulmonary fibrosis (IPF), interstitial lung disease (ILD), asthma, chronic obstructive pulmonary disease (COPD), cystic fibrosis, bronchiectasis, sarcoidosis, silicosis, and other non-COVID-19 viral or bacterial respiratory infections.

**Figure 1:**
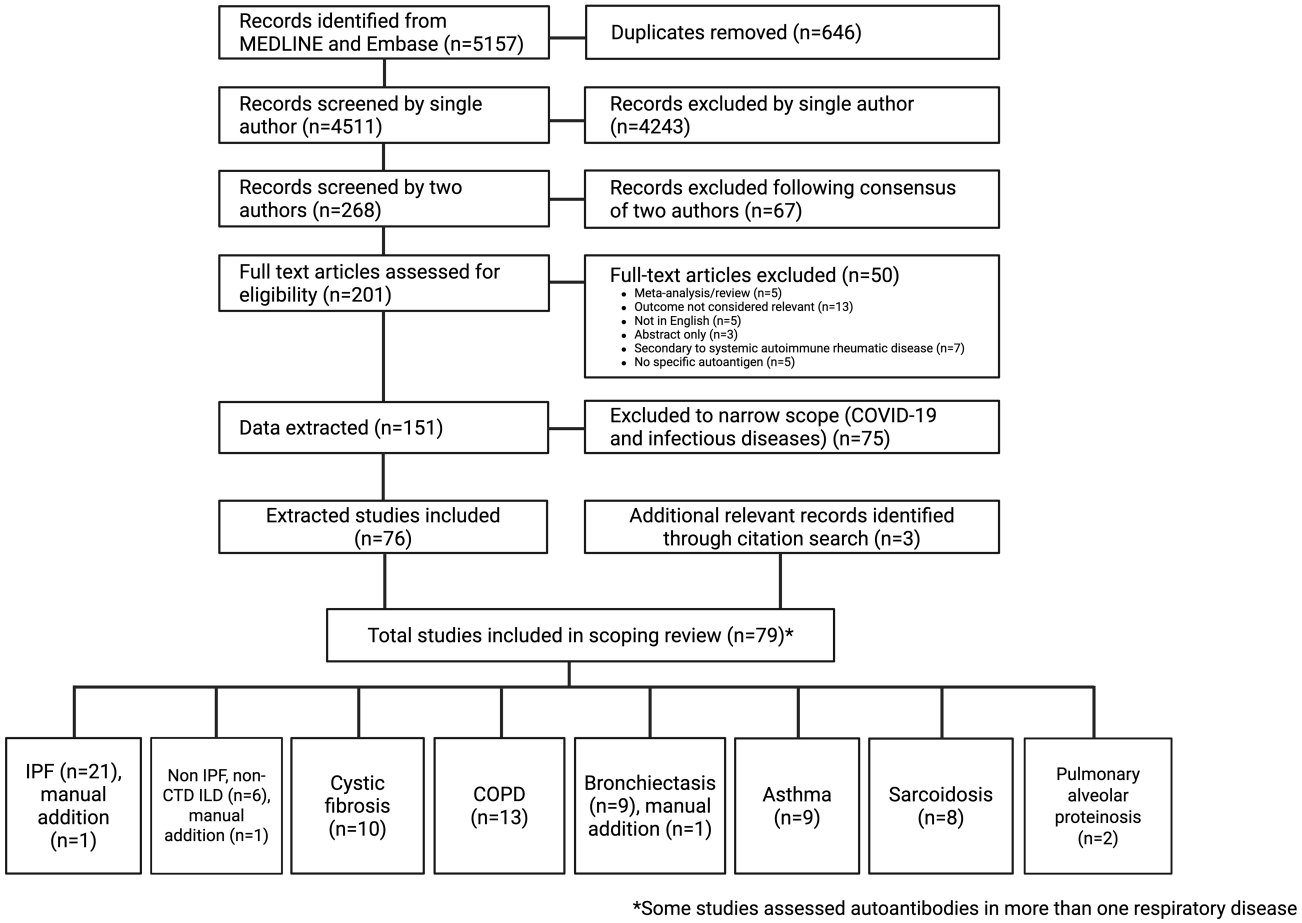
PRISMA flow diagram summarising study selection for the scoping review. MEDLINE and Embase were searched from the start of the database through 13 September 2024. After duplicate removal, 4511 records were screened by a single author and 268 records were screened by two authors. A total of 201 full-text articles were assessed for eligibility, with 79 studies being included in the final synthesis, which included three identified through manual citation review. Categories at the bottom represent overlapping disease groups if some studies reported meaningfully on more than one disease, therefore the totals may exceed 79.

Following full-text assessment and preliminary data extraction it became evident that COVID-19 in particular and to a lesser extent other infectious-related studies dominated the literature and introduced substantial conceptual heterogeneity and bias. To maintain conceptual coherence, the scope was refined *post hoc* to exclude COVID-19 and infectious diseases. This refinement is clearly documented in the PRISMA flow diagram (**Figure 1**). The final review therefore represents a focused synthesis of autoantibody findings across chronic non-infectious respiratory diseases. A PICOS-style framework summarises the eligibility criteria for this scoping review (**Table 1**).

**Table 1:** PICOS-style summary of eligibility criteria used to structure the scoping review question and define study inclusion parameters.

Component	Definition
Population	Adult patients with primary, chronic, non-infectious respiratory diseases including idiopathic pulmonary fibrosis, non-IPF interstitial lung diseases, cystic fibrosis, COPD, bronchiectasis, asthma, sarcoidosis, silicosis
Intervention/exposure	Presence or measurement of autoantibodies in any sample type
Comparator	Healthy or disease controls where applicable for case-control studies
Outcomes	Reported prevalence, presence or levels of autoantibodies
Study design	Observational studies including case-control or cohort study designs; minimum 30 in each arm or 40 total cases in cohort studies respectively

The databases MEDLINE and Embase were searched through to 13 September 2024 using a defined search strategy (**Supplementary File 1**). Duplicates, non-English, non-peer reviewed, and animal-only studies were excluded. Minimum sample sizes were set at 30 per group for case-control studies and 40 for cohort studies. Case control and cohort studies were filtered using strategies outlined in the BMJ Practice Search design filters (9).

Studies investigating autoantibodies in primary respiratory diseases were included, and those examining respiratory diseases that occur secondary to systemic autoimmune rheumatic diseases (SARDs) were excluded to maintain the focus of the review on respiratory-predominant pathology. In the case of bronchiectasis secondary to other conditions, given the strong clinical and immunology overlap with SARDs and limited availability of idiopathic cases, these studies were included. Notably, classification boundaries were not always clearly defined across studies with some cohorts labelled as IPF or IPF with autoimmune features (IPAF) that included participants with low titer or isolated autoantibody positivity without a confirmed SARD. In these cases, studies were retained when the majority of patients had a primary respiratory disease or where their clinical phenotype did not fulfil criteria for a systemic autoimmune diagnosis. This dataset therefore reflects studies describing low-level or isolated serologic autoimmunity in otherwise idiopathic or non-SARD related respiratory disease.

Initial title and abstract screen were performed by one reviewer (KLC) to exclude papers that were deemed irrelevant, abstract-only or were not written in English. A second title and abstract screening stage with two reviewers (KLC and MB) was performed, in which disagreements were further arbitrated. Full-text review was performed by two reviewers (KLC and GK). Data extraction was performed by KLC using a structured charting template in Covidence. This tool captured key study characteristics including country, population tested, autoantibodies tested, methods and pertinent findings. Extracted data was transferred to Microsoft Excel for synthesis and grouping by disease focus. Final data was recorded and analysed by KLC.

## Results

A total of 5157 articles were extracted. After duplicate removal and initial abstract and title screen, 268 articles were assessed by two reviewers and reduced to 201 articles for full text review and finally, 151 for data extraction. Seventy-six studies were included for the review. Three additional studies were deemed relevant with novel information, leading to a total of 79 studies included in this scoping review (**Figure 1**).

The number of studies per disease, key autoantibodies tested, testing methods and any functional validation studies are summarised in **Table 2**. A more detailed breakdown per study can be found in **Supplementary File 2**. Across all disease categories, reported autoantibodies were predominantly supported by associative or biomarker-level evidence. Functional validation studies were sparse and inconsistently reported across diseases and autoantibodies. Consequently, most findings remain at the level of association rather than established mechanistic involvement.

**Table 2:** Summary of studies, key autoantigens assessed, techniques used and functional studies performed.

Disease	Studies (n)	Autoantigens assessed	Common assays	Functional evidence
Idiopathic pulmonary fibrosis (IPF)	22	Routine (ANA, ENA, RF, CCP, dsDNA, MPO, PR3); structural (periplakin, vimentin); stress/innate (HSP70, HSP72, IFNs); discovery-based (MX1, UBE2T, annexin I)	IIF, nephelometry, Bioplex, ELISA, blot assays, immune-precipitation, Gyros, protein microarray, SEREX	Reported for periplakin, HSP70 and HSP72
Non-IPF idiopathic interstitial pneumonias	7	Routine (ANA, ENA, myositis, RF, CCP); lung-specific/autoimmune (BPIFB1); Discovery-based (CDHR5, MX1)	IIF, line blot, ELISA, radioimmunoassay, PhIP-Seq, protein microarray	Minimal; BPIFB1 reported mouse models and T cell transfer studies supporting immune involvement but not supporting direct antibody pathogenicity
Cystic fibrosis	10	Infection-related (BPI (+/- pseudomonas aeruginosa)); citrullination/NET-associated (RF, CCP, carbamylated proteins); Miscellaneous (ASCA, glycans)	ELISA, immunoblot, RIA, IIF	One study assessing NET induction for BPI, otherwise minimal
Chronic obstructive pulmonary disease	13	Routine (ANA, ENA, dsDNA, MPO, PR3, TPO, smooth muscle, mitochondrial, LKM); infection-associated (BPI); structural (CK18, CK19, elastin, N-Ac-PGP, αB-crystalline/HSPB5); cell stress and immune response (GRP78, lactoferrin); Discovery (broad range)	ELISA, IIF, immunoblot, multiplex bead array, protein microarray, HuProt	Macrophage stimulation assay for GRP78, otherwise limited
Bronchiectasis	10	Routine/systemic autoimmunity (ANA, ENA, AMA, aPLs, smooth muscle, parietal cell, MPO, PR3, RF, CCP); otherwise citrullinated proteins (carbamylated proteins, calreticulin); infection-related (BPI)	IIF, Phadia, ELISA	Not reported
Asthma	9	Epithelial (CK8, CK18, CK19, periplakin, collagen V); routine (ANA, RF, CCP, MPO, PR3, ANA, ENA, TPO, TTG); airway-localised (EPX); cytokines (IL-33)	ELISA, immunoblot, IIF, line blot, multiplex bead array, luciferase immunoprecipitation	IL-33 neutralisation assay and mice models, otherwise limited
Sarcoidosis	8	Routine (ANA, ENA, dsDNA, RF, CCP, β2GP1, cardiolipin, TPO, thyroglobulin, ANCA); citrullination-related/structural (citrullinated vimentin, mutated citrullinated vimentin); protein array-derived (CRP, elastin, heparin, MBP, BPI, fibrinogen IV, hemocyanin, histones, Matrigel, prothrombin protein, Sm, TPO, vimentin); discovery-based (ZNF688, ARFGAP1, MRPL43, NCOA2, NELF-E, cofilin μ, chain A)	ELISA, nephelometry, IIF, protein microarray, suspension bead array, planar protein array, prior PhIP-Seq	Not reported
Other (autoimmune pulmonary alveolar proteinosis)	2	GM-CSF	ELISA	Not done as established pathogenic role

**Figure 2:**
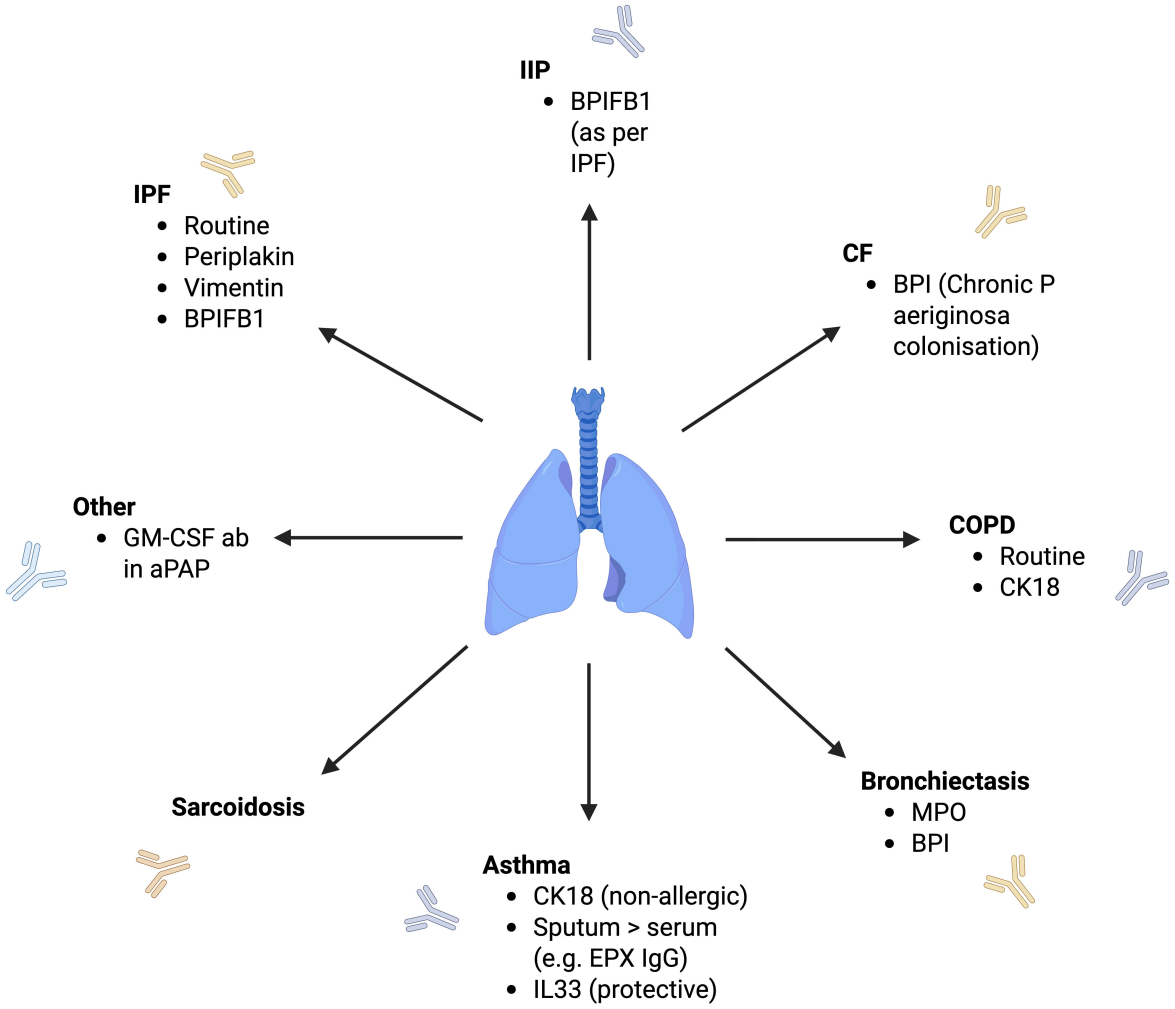
Summary of key autoantibodies reported across chronic non-infectious respiratory diseases. Representative autoantibodies of emerging interest are shown for each disease that warrant further investigation. For sarcoidosis, multiple candidate autoantibodies have been reported across studies; however, none has demonstrated consistent replication or clear predominance. Therefore, no single autoantibody is highlighted. aPAP, autoimmune pulmonary alveolar proteinosis; BPI, bacterial permeability-increasing protein; COPD, chronic obstructive pulmonary disease; CF, cystic fibrosis; CK18, cytokeratin 18; EPX, eosinophil peroxidase; GM-CSF, granulocyte-macrophage colony-stimulating factor; IPF, idiopathic pulmonary fibrosis; IIP, idiopathic interstitial pneumonia; MPO, myeloperoxidase.

The following results sections groups studies by the disease process. A summary of key emerging autoantibody targets is shown in **Figure 2**.

### Idiopathic pulmonary fibrosis

IPF is a chronic, progressive lung disease characterized by the thickening and fibrosis of the tissue around the alveoli in the lungs, leading to reduced gas exchange and progressive loss of lung function. Twenty-one studies which assessed autoantibodies detected in patients with IPF were included in this review, and one manually included study resulted in a total of 22 reviewed studies.

#### Routine clinical assessments for autoimmune diseases

Ten studies in this review investigated the association of IPF with autoantibodies in routine clinical use. Studies were included if they included patients that were considered to have IPF without systemic autoimmune rheumatic disease (SARD). Studies were excluded if the patients were considered to have an underlying SARD for which the detected autoantibodies were associated. Inclusion therefore was reliant upon classification of IPF as primary or secondary. Included studies used a range of testing methods and a range of cut offs for these antibody tests. This variation included tests in common use such as immunofluorescence testing e.g. anti-nuclear antibody (ANA). When using this method to confine the analysis to primary IPF studies only, a resultant two studies were case-control studies (10, 11) and 8 were cohort studies.

The case control study published by Lee et al. reported virtually no difference between patients with IPF compared to healthy controls having one of any routine diagnostic autoantibodies (22% and 21% respectively) (10). Frequency of ANA ≥1:320 in both groups was 27%. There was a mild increased prevalence of RF positivity ≥60IU/mL in cases (6% versus 0% in healthy controls). Other individual autoantibodies (including anti-cyclic citrullinated peptides (CCP), Smith (Sm), ribonucleoprotein (RNP), Sm/RNP, Scl-70, Jo-1, anti-Ro (SS-A), anti-La (SS-B), double stranded deoxyribonucleic acid (dsDNA), chromatin, ribosomal P, centromere B, proteinase 3 (PR3), myeloperoxidase (MPO) and glomerular basement membrane (GBM)) showed no difference. There was a trend towards longer transplant-free survival in those with positive autoantibodies, but this was not statistically significant (HR 0.43, p=0.11).

Several studies compared autoantibody positive to autoantibody negative groups within IPF. No statistically significant survival difference was noted when accounting for seropositivity in many studies (12–16). Kirgou et al. found no difference in mortality, but did find a slower deterioration of DLCO for patients with autoantibody positivity (16). Song et al. reported no difference of the effect of pirfenidone on IPF progression and no difference in forced vital capacity (FVC) or diffusing capacity for carbon monoxide (DLCO) after one year of follow up based on seropositivity grouping (17). Interestingly, Izhakian et al. found high titre ANA positive patients (≥1:160, n=41) had higher mortality rates (68.3% vs 54.1%, p<0.001) when compared to ANA low titre or seronegative patients (n=120). In Cox proportional hazards model, high ANA titres when treated as a dichotomized variable remained strongly associated with case-specific mortality (HR 2.25, 95% CI 1.14-4.20, p=0.02) however the use of dichotomous ANA is clinically questionable (18).

In one study by Ghang et al., 138 autoantibody positive IPF patients and 374 seronegative IPF patients were compared and mortality in the whole cohort was similar whether patients had immunomodulatory therapy or not (14). In the 138 autoantibody positive patients specifically, there was a higher risk of mortality at 5 years with the use of glucocorticoids (HR = 2.21, p=0.019), whereas the use of non-glucocorticoid immunomodulators demonstrated a beneficial effect (HR = 0.31, p=0.002). Although this substantial difference may be caused by small sample size after treatment splitting, this effect needs further investigation.

Specifically focusing on ANCA, Hozumi et al. reported on the comparison between 16 PR3-ANCA interstitial pneumonia cases (with 3 being PR3-ANCA+ IPF), and 94 ANCA negative IPF controls (19). They found no differences in the 5-year survival rate. There was a slightly lower forced expiratory volume (FEV1)/FVC ratio in the PR3-ANCA+ group than negative group (77% vs 84% respectively, p=0.001). None of the PR3-ANCA+ IIP patients developed ANCA vasculitis during the study (median follow up period of 1.8 years, range 0.45-8.5). In another four studies, MPO or PR3 positivity ranged between 0.8-13.4% of cases (10, 14, 15, 17). Overall, the significance of ANCAs in IPF, in which primary vasculitis has not been diagnosed, is questionable.

CCP antibodies were assessed by Solomon et al. whereby CCP IgA autoantibodies were the predominant finding in IPF overall (21.3% in IPF versus 5.6% healthy controls, p<0.01) which may suggest a mucosal origin for these antibodies. No differences were seen in FVC or DLCO between autoantibody-positive or negative IPF patients (11). A trend to longer survival for patients with IPF was associated with the absence of anti-CCP IgA autoantibody-negative patients, when compared to anti-CCP IgA autoantibody-positive patients but this did not reach statistical significance. Other studies did not find a high prevalence of CCP autoantibodies, ranging from 1-2.6% (10, 12, 17). RF was more variable with prevalences ranging from 6-45% (10, 12, 14–17). Anti-dsDNA IgG autoantibodies were infrequent, with frequencies between 1.5-6.6% (10, 12, 14, 17). The anti-dsDNA autoantibody prevalence as similar in cases compared to healthy controls in the case control study (10).

Overall, there is insufficient evidence to suggest a consistent difference in the prevalence of routine diagnostic autoantibodies in IPF patients without a clearly defined SARD compared to controls, nor an overall mortality difference related to seropositivity. However, one study reported a possible link between ANA seropositivity and risk of mortality, warranting further investigation.

As previously discussed, there may be a potential selection bias of studies in which individuals were classified as ILD with secondary SARD based on autoantibodies even in the absence of other clinical autoimmune features. Such studies would have been excluded, potentially leading to the inclusion of studies of patients only with low-level autoantibodies. For example, the relevance of ANCA to lung disease can be difficult to ascertain in individual cases. Those with an ANCA as well as lung disease may have been categorised as ANCA-associated vasculitis by authors even without other systemic manifestations and therefore inadvertently excluded from selection in this review.

#### Periplakin

In 2011, Taille et al. discovered the novel finding of anti-periplakin (PPL) autoantibodies in 16/40 (40%) of patients with IPF compared to 0/40 (0%) of the healthy controls population (20). PPL is expressed in the normal and fibrotic human lung, on the surface of bronchial and alveolar epithelial cells. Anti-PPL autoantibodies were associated with more severe disease symptoms and with lower pulmonary function. In 2023, Koether et al. reported on the findings of anti-PPL autoantibodies in 12/46 (26%) IPF patients versus 3/36 (8%) of their age-matched but not sex-matched controls (21). PPL-binding cells were detected in lung tissue of IPF patients with circulating anti-PPL autoantibodies after incubation using biotinylated PPL. These cells were also found to express CD138, suggesting they were plasma cells, potentially identifying an autoantibody reservoir. In a separate IPF cohort, they described that the clinical significance of anti-PPL autoantibodies, of which 42/163 (26%) of IPF patients were positive, was correlated with a reduced major event-free survival rate as compared to individuals with IPF who were seronegative (121/163, 74%). Major event free survival for anti-PPL autoantibody-positive patients was at a median of 22.3 months compared to 30.3 months in anti-PPL autoantibody-negative patients. In addition, a trend of increased fibrosis was detected in *in vivo* mouse models treated with anti-PPL IgG. These models also showed a minor increase of CXCR5+ fibrocytes in both blood and lung tissue, which parallels some human data. Overall, these findings, which have been reproduced across multiple cohorts, suggest that a subset of IPF is potentially related to autoimmunity, where an antigen at the disease site is driving the appearance of autoantibodies associated with worse clinical outcomes. It is unclear if these autoantibodies are pathogenic.

#### Vimentin

Li et al. reported the assessment of anti-vimentin autoantibodies in two cohorts. The discovery cohort consisted of 102 IPF patients, 50 controls, and 46 COPD patients. This was followed by a replication cohort of 70 IPF patients and 60 controls (22). The prevalence of anti-vimentin IgG was higher in IPF patients than in normal controls in both cohorts. Furthermore, these reactivities were associated in the first cohort with HLA-DQβ1*02 and DRβ1*11 but was inversely associated with HLA-DRβ1*1501, with the latter finding also being validated in the replication cohort. Furthermore, the antibodies were inversely correlated with lung function. Serious adverse outcomes were most frequent amongst patients that had the highest anti-vimentin autoantibody levels (HR 2.5 and 2.7, p=0.012 and 0.006 respectively in each cohort). The authors observed that circulating CD4+ T-cells isolated from IPF patients exhibited dose-dependent proliferation in response to vimentin stimulation, which was abolished by treatment blocking HLA-DR. Similarly, they found that vimentin induced *in vitro* autoantibody production in IPF peripheral blood mononuclear cell (PBMC) cultures, and these responses were HLA class II-dependent. Collectively, their findings revealed that vimentin can be a potent autoantigen that stimulates both B and T cell responses and that levels were higher in IPF patients, were HLA-DR biased and were associated with poorer transplant-free survival over 2 years.

Le Guen et al. assessed for anti-mutated citrullinated vimentin autoantibodies (anti-MCV) in IPF patients (23). They found a high frequency (29/86, 34%) of patients who were positive for anti-MCV, albeit at low titers. There were no clinical associations or differences in transplant-free survival between the anti-MCV antibody positive versus negative groups. This suggests that the citrullination process is not specific to IPF though more research would be needed to draw definitive conclusions.

#### Miscellaneous

We identified a further six standalone studies that each focused on individual autoantibodies that were not investigated in other IPF studies. These included two studies assessing heat shock proteins, and one study each for bactericidal/permeability-increasing fold-containing B1 (BPIFB1); anti-parietal cell antibodies; and type 1 interferon (T1IFN) antibodies.

One study of heat shock protein 70, anti-HSP70 IgG, which correlated with worse outcomes in autoantibody positive patients in terms of FVC and 1 year survival compared to controls. This finding was likely not IPF-specific as was seen in other interstitial lung diseases (24). Another study of anti-HSP72 IgG or IgA found that there was higher total IgG in IPF cases compared to other ILD and healthy controls, and higher IgA in IPF cases and other ILD compared to healthy controls (25). *In vitro* experiments with monocyte-derived macrophages (MDMs) found that anti-HSP72 complexes stimulated macrophages to secrete CXCL8 and CCL18.

Shum et al. reported a novel link between the lung-specific protein bactericidal/permeability-increasing fold-containing B1 (BPIFB1) and clinical ILD after discovering autoantibodies to this antigen in autoimmune polyglandular syndrome type 1 complicated by ILD (26). Although this included patients with IPF, given the heterogeneity of cases and inclusion of non-IPF ILD it will be detailed in the non-IPF IIP section of this review.

Other autoantibodies assessed in studies of IPF included anti-parietal cell autoantibodies, for which clinical significance could not be established (27); and anti-Type I interferon autoantibodies, which were not elevated in IPF patients compared to healthy controls (28).

Arai et al. assessed the frequency and clinical associations of autoantibodies of IgG, IgA and IgM isotypes against myxovirus resistance protein 1 (MX1) in 71 IPF patients (29). They had previously identified anti-MX1 to be an autoantibody detected in the sera of individuals with nonspecific interstitial pneumonia (NSIP) using protein microarrays (30). MX1 is a type of GTPase that is induced by T1IFNs and plays an antiviral role. In this study, they detected 18 (25.4%), 4 (5.6%) and 0 (0%) of IPF patients had elevated levels of serum anti-MX1 IgG, IgA, and IgM, respectively. Survival of 4 IPF patients with higher anti-MX1 IgA autoantibodies was worse than the other patients without elevated anti-MX1 IgA antibodies (using log-rank test, p<0.001), and acute exacerbations also occurred more frequently (log-rank test, p=0.035). These results must be interpreted with caution due to the low numbers of patients with high anti-MX1 IgA autoantibody concentrations. There was no difference in survival or incidence of exacerbation events with anti-MX1 IgG autoantibody concentration.

#### Unbiased discovery-based autoantibody screening technologies

Ujike-Hikichi et al. used a proteome microarray to detect autoantibodies in blood to any of 9483 full-length recombinant proteins, in patients with ILD (in 5 healthy controls, 7 IPF and 2 connective tissue disease-associated ILD (CT-ILD)). The highest signal was for autoantigen ubiquitin conjugating enzyme E2T (UBE2T) in ILD patients. This autoantibody was detected in 7 IPF patients and 2 CTD-ILD patients (31). UBE2T is a human ubiquitin-conjugating enzyme that is important for the ubiquitination process, leading to proteasome-mediated protein degradation and DNA repair. Their findings of anti-UBE2T autoantibodies were confirmed in a larger group of 43 IPF patients and twenty-one fibrosing nonspecific interstitial pneumonia (NSIP) patients versus 61 healthy controls. Median anti-UBE2T autoantibody concentration was higher in the ILD cohorts compared to healthy controls. Furthermore, after two years of follow up they determined retrospectively that patients with progressive ILD had higher anti-UBE2T autoantibody levels compared to non-progressive ILD (339ng/mL versus 216ng/mL, p<0.01). In twelve patients where additional samples were taken, there was no longitudinal change in antibody concentration after 6–12 months. UBE2T protein was also found strongly expressed in lung tissue biopsies from 9 patients with IPF lung tissues but not found in lung tissue biopsies of twelve control patients. UBE2T was detectable in the bronchiole epithelium and regenerative type II alveolar epithelium lining. These findings strongly suggest a difference in anti-UBE2T autoantibodies in cases compared to controls although no functional studies were done.

Kurosu et al. tested sera from 45 IPF patients using Serological identification of Antigens by Recombinant Expression cloning (SEREX), a technique applied to investigate antibodies in a variety of tumour systems and autoimmune disease such as systemic lupus erythematosus and systemic sclerosis (32). They found reactivity to twelve antigens through this screening method, with the most prominently identified antigens being annexin 1 and Bax inhibitor 1. Autoantibodies to annexin 1 were detected in 7/15 (47%) and 8/15 (53%) cases of the sera and BAL materials respectively from patients with acute exacerbation of IPF, and 2/30 (7%) and 3/30 (10%) cases of the sera and BAL materials from stable IPF patients. In contrast, no autoantibodies were found in sera or BAL fluid from 40 healthy controls and non-IPF subjects, and only 10% of sera from patients with Rheumatoid Arthritis (RA) and SLE-related interstitial pneumonia had anti-annexin 1 autoantibodies. Western blot for annexin 1 antigen in BAL samples confirmed that annexin 1 antigen (either full length or cleaved length, 36 kDa or 33 kDa respectively) was detected in all 15 cases of acute exacerbation of IPF. Overall, there appeared to be a signal for annexin 1 autoreactivity in patients with acute exacerbation of IPF but further research is required to validate this in a separate cohort before determining any pathogenic role.

### Other non-IPF idiopathic interstitial pneumonias

This section includes seven studies, including one manually included, that assessed autoantibodies in idiopathic interstitial pneumonia (IIP) beyond IPF alone. Interstitial lung diseases (ILDs) are a large group of chronic respiratory disorders that primarily affect the tissue and space surrounding the alveoli. The hallmark of these conditions is inflammation and progressive fibrosis of lung tissue resulting in declining lung function. These diseases have been classified into major idiopathic interstitial pneumonias (which includes IPF, but also includes idiopathic NSIP (as alluded to above), respiratory bronchiolitis-interstitial lung disease, desquamative interstitial pneumonia, cryptogenic organizing pneumonia and acute interstitial pneumonia), rare idiopathic interstitial pneumonias (idiopathic lymphoid interstitial pneumonia or idiopathic pleuroparenchymal fibroelastosis) or unclassifiable idiopathic interstitial pneumonias (33). Once again we focused on studies that included IIPs without an already known underlying SARD or alternative cause (for example, hypersensitivity pneumonitis).

Bermea et al. showed that ILD patients with antibodies to RNA-binding proteins (SSA, SSB, Sm, RNP) had worse respiratory disease with lower %FEV1 and %FVC but no differences in %DLCO nor transplant-free survival differences (34). Morita et al. identified seropositivity to Ro52 autoantibodies in 17 individuals who had a lower rate of 2-year survival compared to Ro52 seronegative patients, but this small study requires validation (35). Correia et al. assessed anti-CCP antibody titers and ILD frequency in 2030 individuals who had had them tested. In this cohort, higher anti-CCP Ab titers (89.7U/mL vs 28.U/mL) or RF seropositivity (65.2% vs 29.9%) were found in patients with ILD enrolled in compared to those without ILD (36). Zheng et al. assessed a national registry and found seropositive individuals with non-CTD ILD had similar rates of FVC decline compared to seronegative individuals (37). There was no statistically significant difference in duration of survival or rates of progression to lung transplant when comparing seropositive or seronegative non-CTD ILD. Both CCP and RF studies suggest that seropositive individuals may be at higher risk of ILD in either RA or non-confirmed RA, however, in those with CTD-ILD seropositivity does not significantly alter prognosis.

As alluded to in the IPF section, Shum et al. published their novel findings of BPIFB1 autoimmunity in ILD (26). First discovering these anti-BPIFB1 autoantibodies in APS1 patients with secondary ILD, they then discovered the same antibodies in 7/48 (14.6%) of patients with CTD-ILD and 13/108 (12%) of idiopathic ILD, but none in 27 non-ILD lung disease controls matched for age and sex, nor patients with Type 1 diabetes mellitus. Mouse models using the BPIFB9 antigen revealed serum transfer of anti-BPIFB9 antibodies did not induce lung disease, but transfer of BPIFB9-specific T cells into the mice did. Furthermore, thymic engraftment experiments revealed AIRE and central tolerance was important for preventing respiratory disease. Overall, their findings suggest immunoreactivity to BPIFB1, though not specifically through autoantibodies, is associated with autoimmune respiratory disease through negative selection mediated by AIRE in ILD. The autoantibodies are likely a bystander effect that may still be useful for diagnosis and prediction of ILD development.

#### Unbiased discovery-based autoantibody screening technologies

##### Cadherin-related family member 5

Upadhyay et al. performed PhIP-Seq in a large unbiased antibody discovery screen in ILD in 398 cases (80 CTD-ILD, 70 hypersensitivity pneumonitis, 102 IPF, 10 interstitial lung abnormality, 81 other, 28 unclassifiable, 27 unknown CTD-ILD), and 184 controls (138 blood bank, 46 non-ILD controls) (38). They discovered a novel autoantigen associated with ILD, CDHR5, which is a protein involved in cell adhesion. When validating the presence of CDHR5 using radioligand-binding assay, they found that 18 different patients out of 699 total samples taken had elevated autoantibodies. Amongst the 18 patients, 9 had known history of a systemic autoimmune rheumatic disease (SARD), however, the antibodies were also found in patients without SARD. When performing a survival analysis assessing for transplant free survival or death in one cohort (n=250), they discovered that patients with CDHR5 antibodies (n=10) had worse survival (p=0.0044). Given the low numbers of autoantibody positive patients this should be interpreted with caution. Based on an IPF Cell Atlas, they found CDHR5 expression was sparse and where detectable was found in ciliated cells, alveolar type 2 cells, and some hematopoietic cells. Taken together, it suggests that patients with CDHR5 antibodies may represent a small subset of ILD patients with progressive lung disease.

##### MX1

Hamano et al. utilized protein microarray as well as immunoprecipitation on a discovery cohort to discover that anti-MX1 autoantibodies were higher in patients with NSIP (n=8), compared to IPF (n=10) and healthy controls (n=10) (30). This was then confirmed in another cohort of 114 individuals with chronic fibrosing IIPs (n=114), which included IPF (n=19) and non-IPF (n=95), compared to HC, which demonstrated higher optical densities via ELISA for IgG, IgA and IgM autoantibody isotypes in NSIP patients. In a third cohort (IPF n=71, non-IPF n=84), after adjustment of disease stage by modified ILD-gender-age-physiology (ILD-GAP) index, they compared the survival rate between non-IPF patients with (n=20) and without anti-MX1 autoantibodies (n=59). They found that those with anti-MX1 autoantibodies had a better survival rate compared to antibody-negative patients (p=0.037). This study led to further testing of anti-MX1 autoantibodies in IPF, as previously discussed (29).

### Cystic fibrosis

Cystic fibrosis (CF) is a monogenic disease caused by mutations in the *CFTR* gene, that results in impairment of chloride and carbonate ion exchange and leads to dehydrated viscous mucus. The lungs, intestinal tract, and pancreas develop significant pathologies including altered microbial colonization and susceptibility to a range of bacterial infections. A total of 10 studies are included in this section of the review.

#### Bactericidal/permeability-increasing protein

BPI is an anti-microbial protein expressed in the azurophilic granules of neutrophils and is especially potent against gram-negative bacteria such as *Pseudomonas aeruginosa*. Autoantibodies to BPI have been described in autoimmune diseases including inflammatory bowel disease and primary sclerosing cholangitis (39).

We identified four studies that investigated the prevalence and clinical relevance of BPI-ANCA autoantibodies in CF. Lindberg et al. compared BPI-ANCA and *P. aeruginosa* serology with respect to lung function and chronic *P. aeruginosa* colonization and found that lung function better correlated with BPI-ANCA IgA autoantibodies (r=0.44) than with anti-pseudomonal serology (r=0.06-0.35, depending on which pseudomonal antigen was assessed) (39). Long-term outcome analysis also yielded a better ability of BPI-ANCA to predict either death or lung transplantation events with a higher AUC (0.77, p=0.002) compared to pseudomonal serology (AUC ranging from 0.54-0.7).

Skopelja et al. reported the prevalence of anti-native BPI (nBPI) autoantibodies in 16/38 (42%) of adult serum CF samples and 0/50 (0%) in RA controls, whilst anti-carbamylated protein autoantibodies were seen in 40% of CF samples compared to 28% of RA samples (40). CF samples showed autoreactivity to either nBPI or carbamylated fibrinogen (and/or fibrinogen) but not both. The authors described the antibody reactivity to the C-terminus of BPI, and that the cleavage of BPI in *P. aeruginosa*-related NETs occurred in a *P. aeruginosa* elastase-dependent manner. In terms of clinical correlation, a negative correlation was found between anti-C-terminal BPI IgG and FEV1% (r=-0.66, p<0.0001). The anti-native BPI IgG also negatively correlated with FEV1% (r=-0.468, p=0.003). Together they suggest that anti-BPI IgG are found in patients with deteriorating lung function. Higher autoantibody levels were also found in patients who experienced more than 2 pulmonary exacerbations per year.

Theprungisirikul et al. found serum anti-BPI IgG in two separate cohorts of CF patients, paediatric (n=24) and adult (n=32) (41). Only 1/24 of the paediatric patients had anti-BPI autoantibodies, compared to 53% of the adult CF population. A correlation between anti-*P. aeruginosa* IgG and anti-BPI IgG concentrations was identified, suggesting autoreactivity to BPI develops in the context of a humoral immune response to *P. aeruginosa* (p=0.007 and <0.0001 in two separate cohorts). The bronchoalveolar lavage fluid (BALf) of 52 patients were tested for anti-BPI and anti-pseudomonal antibodies, the latter for both IgG and IgA subclasses. Anti-BPI autoantibodies in BALf were restricted to IgA isotype (58% overall positivity rate, seen in both paediatric and adult samples). Anti-pseudomonas antibodies were found in 60% (IgG) and 58% (IgA) of BALf. Overall, this work strongly suggests that anti-BPI autoantibodies develop over time. Antibody development may follow extended colonization by gram-negative bacteria such as pseudomonal species.

Iwuji et al. found serum BPI-ANCA IgG in 9/47 (19%) in CF patients (42). There was no association of change of FEV1 with detection of BPI-ANCA autoantibodies, at baseline or at one year of follow up. However, a significant difference was found in the proportion colonized with *Pseudomonas* (>50%) compared to patients without (18.4%) at both baseline and follow up. This study also shows a strong association of anti-BPI autoantibodies with chronic pseudomonal colonization and anti-pseudomonal antibodies. Anti-BPI autoantibodies may both be a marker of extended colonization of *Pseudomonas* as well as contributing to perpetuating infection by blocking innate immune anti-microbial responses.

#### Miscellaneous

Whilst the presence of anti-BPI autoantibodies in CF has been reported in multiple studies and cohorts producing similar findings, there have also been a variety of other autoantibodies investigated in CF in standalone studies. One study suggested a possible link between anti-saccharomyces cerevisiae antibodies (ASCA) IgA and *P. aeruginosa* colonization (43). Although without strong clinical correlations, another study found that a significant proportion of CF patients were positive for autoantibodies to various glycans (44). Anti-elastin and proline-glycine-proline autoantibodies were assessed but not detected in CF (45). RF, CCP, carbamylated proteins or citrullinated proteins were examined, and a high prevalence of detectable anti-CCP IgA was observed in each of patients with CF (OR 4.4, 95% CI 1.1-18, p<0.05) and patients with RA (OR 43, 95% CI 10-127, p<0.0001) when compared to healthy controls. It is unlikely that this autoreactivity was citrulline-specific as there were no significant differences in reactivity between citrullinated or native peptides (46). Another study assessed circulating anti-dsDNA IgA concentrations and found them to be higher in CF compared to controls, with an association with impaired lung function but without any other reported clinical or demographic covariates (47). A further study assessed anti-peptidyl arginine deiminase 4 (PAD4), an enzyme that is required for NET formation, which was detected in patients with CF. The presence of these autoantibodies was correlated with lower FEV1%, and *Pseudomonas* in the sputum (48).

### Chronic obstructive pulmonary disease

COPD is a group of progressive lung conditions, primarily emphysema and chronic bronchitis, with increased resistance to airflow which is not fully reversible. A possible autoantigen-autoantibody connection has been hypothesized as a contributory pathogenic mechanism. We summarise 13 studies that sought an association between COPD and autoantibodies.

#### Routine clinical assessments for autoimmune diseases

Four studies assessed the frequency and clinical utility of routine diagnostic autoantibodies. As described above in other studies of antibodies associated with other respiratory diseases, diagnostic titre cut-offs varied across studies. Two studies reported a higher frequency of positive ANA in COPD patients compared to healthy controls, based on indirect immunofluorescence (IIF) at different cut-offs (49, 50). Liang et al. reported two studies from 2019 and 2020 in which their group used multiplex bead array to assess for antibodies to Extractable Nuclear Antigens (ENA) in serum and sputum, identifying four stable COPD subgroups using hierarchical clustering based on principal component analysis (51, 52). The authors reported that sputum autoantibodies correlated better with disease exacerbation than did serum autoantibodies. Whilst promising, these require further studies to validate the presence of autoantibodies in COPD sputum.

#### BPI

Tian et al. investigated the presence BPI autoantibodies in COPD (53). In a cohort of 126 patients with chronic COPD and Pseudomonas colonization, 59/126 (46.8%) of patients were positive for BPI-ANCA. In a following study, the authors validated the findings in an independent cohort, with BPI-ANCA autoantibodies found in 104/216 (48.2) of COPD patients with Pseudomonas colonization versus only 6/244 (2.5%) of individuals with Pseudomonas infection without COPD, or 0% of healthy controls (54). In both studies they found BPI-ANCA positive patients had worse pulmonary function tests as defined by FEV1% and FEV1/FVC in patients with BPI-ANCA autoantibodies compared to patients without detectable autoantibodies. In the second of these two studies, BPI-ANCA positive patients had elevated levels of inflammatory markers such as TNF-α, IL-6 and IL-1β. No functional studies were performed to determine if the detected antibodies were pathogenic.

#### Miscellaneous

There were six individual studies that each investigated possible unique autoantibodies or used a protein array to assess a panel of autoantigens, but these have not been validated in separate cohorts/studies. These included: αB-crystalline (HSPB5) autoantibodies, found to be higher in patients with COPD and/or acute respiratory tract infections compared to healthy controls but without association with severity (55). Anti-elastin and PGP autoantibodies, which were not enriched in samples from patients with COPD (45), or anti-glucose regulated protein 78 (GRP78) autoantibodies which were found to be more frequent in smokers in general compared to non-smoke-exposed controls, and also to be associated with reduced bone density (56).

In addition, Xiong et al. assessed anti-IgG, A and M antibodies to cytokeratin 18 and 19 in COPD patients as well as a mouse model (57). Cytokeratin (CK) proteins are cytoskeleton proteins located in the cytoplasm. Of more than 20 known CKs identified in epithelial tissues, CK18 and CK19 are expressed primarily in bronchial and alveolar epithelial cells. This study found increased circulating levels of IgG, A and M autoantibodies against CK18 and CK19 in a large cohort of 228 COPD patients compared to 136 healthy controls. Furthermore, levels of four of the six antibodies were positively associated with disease severity, with the exception of anti-CK19 IgG and anti-CK18 IgA, suggestive of an association with disease development. The authors observed antibody levels were higher in mice that were exposed to cigarette smoke when compared with antibody levels in mice exposed only to ambient air. Histological studies also demonstrated antibodies in the damaged lung tissue of mice.

Shindi et al. performed a protein microarray to detect IgM or IgG antibodies to 33 antigens felt to be highest yield based on prior literature (58). These included various microbial antigens, intracellular and cytoplasmic proteins and collagens. After setting the cut-off of positivity based on 10 healthy controls but with a >10-year smoking history, different combinations of IgG and IgM antibodies to a range of autoantigens were found. The authors reported a mean number of IgG autoantibody reactivities as 3.5 in moderate COPD, 2.6 in severe COPD and 4.5 in very severe disease, whilst the mean numbers were 3.2, 3.1 and 4.8 for IgM autoantibodies. There was a distinction between autoantigens principally recognized by IgM or IgG autoantibodies, for example, IgG autoantibodies were found to histone and Scl-70, whilst IgM autoantibodies were found to CENP-B and La/SS-B. IgM antibodies are the first antibody class (isotype) to develop in an B cell response to an antigen. B cells require a germinal centre-mediated process to switch antibody class from IgM to IgG. Further research is required to understand the clinical relevance of autoantibody class-switching in COPD.

Cass et al. used a bead-based array platform with 124 nuclear and tissue autoantigens to investigate for IgG and IgM antibodies in matched sputum and sera in two cohorts (one from Europe/North America and one from China) (59). The cohort consisted of 141 COPD patients, 55 asymptomatic smokers and 28 healthy controls. Patients included were graded for lung disease severity using the Global Initiative for Chronic Obstructive Lung Disease (GOLD) grading. Total median serum but not sputum autoantibodies were associated with worsening airflow obstruction, though only in GOLD I and GOLD III subjects compared to controls in the Chinese cohort, making it inconsistently associated with severity. The authors found that anti-elastin autoantibodies in serum were increased in COPD patients in the Chinese cohort. In contrast to Liang et al. (51, 52), they found serum autoantibodies were more abundant than sputum autoantibodies. Overall, their analysis observed a limited difference between sputum and serum antibodies in COPD, asymptomatic smokers and healthy controls in contrast to other studies. The authors considered that the broad assessment of multiple antigen specificities may better demonstrate a lack of autoimmune processes in COPD compared to singular specificities and so questioned the autoimmune hypothesis in COPD.

#### Unbiased discovery-based autoantibody screening technologies

Ma et al. performed an unbiased proteome microarray using HuProt of more than 19000 proteins on a discovery cohort of 5 COPD patients and 5 age- and sex-matched smoking controls (60). Compared to non-COPD smokers, patients with COPD had a distinct autoantibody profile compared to healthy controls. In general, many IgG autoantibodies were found against neutrophil granule proteins. High titres of IgG autoantibodies in COPD patients were found that bound to peptides of extracellular origin, whilst low titres were found binding to peptides of intracellular origin. Conversely, high titers of IgM autoantibodies were enriched to nuclear peptides and functionally related to smooth muscle contraction, whilst low titres were enriched to peptides found in the cytosol, cytoplasm and nucleoplasm. Ma et al. then performed a validation study on 144 COPD cases versus 101 asthma and 124 healthy controls, for IgG autoantibodies against lactoferrin, a neutrophil autoantigen previously described in other autoimmune diseases with antimicrobial properties (61). It was confirmed that COPD patients had a significantly higher frequency of lactoferrin IgG autoantibodies compared to the controls using immunoblotting. This study is likely to suffer from biasing effects of a very small discovery cohort, which is likely to lead to a substantial amount of false negative and false positives. Although the targeted analysis in the large secondary validation accounts for false positives it cannot identify the false negatives missed in such a small discovery cohort given autoantibodies frequently occur in less than 20% of a heterogenous disease.

### Bronchiectasis

Bronchiectasis is a heterogenous chronic respiratory disease in which the airways are dilated, leading to recurrent infections, further inflammation and airway damage. Bronchiectasis can be secondary to CF, recurrent infections, autoimmune diseases, or, in nearly 40% of cases, exists without an identified cause (62). Ten studies were identified that assessed the presence of autoantibodies in non-CF bronchiectasis, including one study which was added following the initial search. In this section, autoantibodies in bronchiectasis related to an underlying primary disease were included alongside studies of bronchiectasis without an identified underlying cause, as few studies assessed autoantibodies in the latter category.

#### Autoantibodies associated with systemic autoimmunity

##### ANA, ENA, SMA

Soto-Cardenas et al. assessed the frequency of routine diagnostic autoantibodies in bronchiectasis as part of primary Sjögren’s syndrome (pSS) (63). As background, most patients with Sjögren’s syndrome are found to have detectable ANA, and detectable antibodies are within the classification criteria of this syndrome. In this study, 39 of 40 (97%) of patients with pSS and bronchiectasis had positive ANA compared to 32/37 (86%) of the control group of pSS without bronchiectasis (p=0.10). Anti-smooth muscle antibodies were more frequent in the pSS-bronchiectasis group (32/39, 82%) compared to the pSS no-bronchiectasis group (21/35, 60%, p=0.043). Sjögren’s syndrome antibodies were less frequent in the bronchiectasis group (11/41, 27% versus 20/37, 54%, p=0.022) as were elevated serum gamma globulins. The antibody differences found in this cohort may be confounded by the older mean age and low frequency of fulfilment of the 2002 pSS criteria in their cohort.

##### ANCA, MPO or PR3

Three studies reported on the frequency of ANCA, MPO or PR3 antibodies in bronchiectasis in association with underlying ANCA-associated vasculitis. Neel et al. reported on 58 French patients with vasculitis, 30 with microscopic polyangiitis and 28 with granulomatosis with polyangiitis (64). Overall, MPO-ANCA and PR3-ANCA were reported in 39 (67.2%) and 19 (32.8%) patients respectively. Bronchiectasis was found in 22 patients (37.9%), all of whom had anti-MPO-ANCA, meaning that 22/39 (56.4%) MPO autoantibody-positive individuals had bronchiectasis. MPO-positive patients with bronchiectasis were more likely to have peripheral nerve involvement (54.5% vs 17.6%, p=0.019) and less frequent renal involvement than MPO-positive patients without bronchiectasis. No difference in survival nor pulmonary infection was found in those with or without bronchiectasis in this MPO-positive subgroup.

Ren et al. assessed ANCAs in a Chinese cohort of 48 patients with ANCA-associated vasculitis and bronchiectasis and compared these with a control group of 164 patients with vasculitis but without bronchiectasis (65). They found that anti-MPO autoantibodies were more prevalent in patients with bronchiectasis compared to those without bronchiectasis (93.8% vs 77.4%, p=0.011), whilst the prevalence of anti-PR3 autoantibodies was lower in those with bronchiectasis compared to those without bronchiectasis (4.2% vs 21.3%, p=0.006). No significant differences in ANA and RF autoantibodies were found between the groups. Patients with anti-MPO autoantibodies were more likely to have bronchiectasis (45/172, 26.2%) and those with bronchiectasis were mostly anti-MPO autoantibody positive (45/48, 93.75%). These studies strongly suggest that MPO autoantibody positivity is more associated with bronchiectasis than PR3 autoantibody positivity.

Lhote et al. found that within a group of 61 adults with AAV (27 MPA, 27 GPA, 7 EGPA), 39/61 (64%) had MPO-ANCA, and 13/61 (21%) had anti-PR3 antibodies, whilst ANCA lacked specificity in six or was absent altogether in five (66). The cohort was grouped into those patients in which bronchiectasis was diagnosed before (n=25, 41%) or after vasculitis (24, 39%), or simultaneously (12, 20%). MPO positivity was more frequent in the bronchiectasis-then-vasculitis group, and this group also had an increased risk of mortality (7/25, 28%) than the vasculitis-then-bronchiectasis group (2/36, 6%, p=0.03). As this is a small number of patients, further studies are required to confirm these observations.

##### CCP, RF, or citrullinated peptides

Five studies assessed frequency of antibodies to CCP, RF and/or antibodies to citrullinated peptides in patients with bronchiectasis. Quirke et al. assessed RF, antibodies to CCP2 and antibodies to citrullinated peptides in patients with bronchiectasis alone, patients with bronchiectasis and RA, and patients with RA but without lung disease, patients with asthma and healthy controls (67). They found that RF, CCP2, citrullinated alpha-enolase peptide (CEP1), and citrullinated fibrinogen were all increased in frequency in bronchiectasis patients with RA compared to all other cohorts, with patients with RA but without lung disease having the second highest frequency. However, they found this response not citrulline-specific as they found cross-reactivity in absorption studies in addition to increased frequency of antibodies to the arginine-containing control peptides (e.g. arginine-containing α-enolase peptide 1, REP-1). Whether this relates to bronchiectasis directly, or rather to the severity of RA leading to bronchiectasis as a co-morbidity is unclear and would perhaps be better distinguished by assessing autoantibodies in alveolar lavage fluid.

Janssen et al. (also discussed in the CF section) studied a cohort of bronchiectasis patients (n=80) and compared them to RA without lung disease (n=86) and healthy controls (n=36) (46). Seropositivity for CCP-IgG in bronchiectasis was 24% compared to 86% in RA and 5.6% in healthy controls, whilst CCP-IgA was found in 10% of bronchiectasis patients compared to 74% in RA and 8.3% in healthy controls. IgM RF was found in 7% of bronchiectasis patients, 74% of RA and 2.8% of healthy controls, whilst IgA RF was increased in 24% of bronchiectasis patients, 50% of RA and 2.8% of healthy controls. Similar to CF, %FEV1 was significantly worse in bronchiectasis patients who were seropositive for IgA RF. Anti-carbamylated protein autoantibodies were not different in bronchiectasis compared to healthy controls, and neither was IgG against various citrullinated proteins. Overall, there was higher prevalence of these autoantibodies in bronchiectasis patients to healthy controls but unsurprisingly less than in RA without lung disease.

Clarke et al. investigated antibodies to citrullinated peptides in patients with bronchiectasis but without RA (n=122) and patients with bronchiectasis and RA (n=52) versus various control groups (healthy controls, n=79, asthma, n=87, RA, n=50) (68). Calreticulin (CRT) and citrullinated calreticulin (citCRT) are implicated in RA. CitCRT binds to RA shared epitopes on HLA-DR and triggers inflammation in adjacent cells. This study found that bronchiectasis without RA, bronchiectasis with RA, and RA consistently had higher autoantibodies to citrullinated proteins compared to healthy controls. 18% of bronchiectasis patients had anti-native calreticulin autoantibodies and 35% had anti-citrullinated calreticulin autoantibodies. 19.6% of bronchiectasis with RA sera were anti-calreticulin autoantibody positive, and 58% were positive for anti-citCRT autoantibodies. No RA alone patient without lung disease had anti-CRT autoantibodies, but 37% of these patients had anti-citCRT autoantibodies. When subtracting for anti-native CRT from anti-citCRT antibodies, IgG antibodies specific for citCRT were observed in: 51% of RA alone, 47% of bronchiectasis with RA, 25% of BR patients and only 4% of controls. Overall, the data suggests that there may be loss of tolerance to citCRT, giving rise to autoantibodies to this peptide in bronchiectasis, bronchiectasis with RA and RA compared to healthy controls, and that this citrullinated peptide of CRT may be the predominant autoantigen, not native CRT in bronchiectasis patients. This study does not determine if there is a direct association of autoantibody with bronchiectasis.

Two further studies assessed the seropositivity of CCP and RF in patients with bronchiectasis with RA compared to patients with RA but without lung disease (69, 70). In bronchiectasis with RA vs RA patients, higher frequencies of CCP and RF in those with bronchiectasis were found compared to RA patients without lung disease (69). McDermott et al. found that the OR of seropositivity in RA patients with bronchiectasis compared to RA alone was 3.96, for RF positivity 4.40, and for CCP positivity 3.47 (70). Furthermore, they described that in bronchiectasis patients, higher RF titers were correlated with lower %FEV1 (-0.38, p=0.015), %FVC (p=-0.32, p=0.045). There was a statistically significant correlation between higher CCP antibody titers and lower %FVC (p=-0.38, p=0.025) but not between CCP antibody titers and %FEV1 or FEV1/FVC. Collectively these findings suggest that whilst there was an increased frequency of CCP and/or RF in bronchiectasis patients, whether it is citrulline specific is inconclusive. Furthermore, it can be concluded that seropositivity is more frequent in patients with RA who have bronchiectasis and emphasize the important and complex interactions between airway inflammation and RA but again whether this relates to bronchiectasis or the disease course of RA itself impacting other organs remains unclear.

#### Autoantibodies associated with chronic infection

##### BPI

Skopelja-Gardner et al. reported anti-BPI autoantibodies in two cohorts of bronchiectasis at a frequency of 52% (n=16) and 56% (n=42) respectively (71). However, although they state the method used was ELISA, it is not clear how detected antibodies were considered positive, or what cut-off was used. As seen in studies of patients with CF, these authors also found that there was a strong association between anti-BPI autoimmunity and anti-*P. aeruginosa* IgG titers in patient sera (n=16, p=0.003). Furthermore, another longitudinal study of 34 more bronchiectasis patients demonstrated the autoantibody concentrations to BPI and *P. aeruginosa* changed in the same direction over two consecutive visits.

Overall, there is a surprising lack of investigation into novel autoantibodies in bronchiectasis despite a subset of patients with this disease having a known autoimmune etiology. All studies of bronchiectasis were undertaken not as an unbiased investigation of the disease but rather targeting bronchiectasis in the context of other known autoimmune diseases and their known autoantigens. Therefore, most studies focused on routine assessment of standard clinical autoantibody diagnostic tests linked to other conditions. There is a gap in our knowledge around autoantibodies and bronchiectasis, including idiopathic forms of the disease.

### Asthma

Asthma is a chronic respiratory disease where airways are usually reversibly narrowed leading to dyspnoea and wheeze. Nine studies were included in this section.

#### Cytokeratins

Cytokeratins are cytoskeletal structural proteins that are expressed only in epithelial cells. They can be found in bronchial and lung alveolar epithelial cells, but also epithelial cells lining most other organs (72). Circulating anti-cytokeratin IgG autoantibodies were reported in IPF and autoimmune hepatitis, whilst circulating IgA antibodies to Cytokeratin 18 (CK18) have been reported in patients with RA.

Three studies assessing anti-cytokeratin autoantibodies in asthma are included in this review. Both Nahm and Mohammad et al. found that anti-CK18 IgG autoantibodies were higher in non-allergic asthma compared to allergic asthma or healthy controls (72, 73). However, the former did not assess severity, and the latter did not find a statistically significant increased frequency of antibodies in more severe asthma compared to milder asthma. Ye et al. assessed the frequency of antibodies to various cytokeratins (8, 18 and 19) in patients with aspirin-intolerant asthma (n=79), in comparison to each of patients with aspirin-tolerant asthma (n=61) and healthy controls (n=88) (74). The authors found both of anti-CK18 and anti-CK19 autoantibodies to be more frequent in aspirin-intolerant asthma (13.9% for anti-CK18 and 17.7% for anti-CK19 antibodies) compared to healthy controls (p=0.014 and p=0.02 respectively). In addition, significant correlations were found between CK18 and CK19 IgG autoantibodies and airway hyper-responsiveness measured by PC20 methacholine challenge in each group.

#### Routine clinical assessments for autoimmune diseases

Ye et al. also assessed prevalence of detectable ANA and did not find a difference in ANA prevalence between either patients with aspirin-intolerant asthma (n=79), patients with aspirin-tolerant asthma (n=61) nor healthy controls (n=88) (74). In contrast, Tamai et al. used IIF to show that 10% of 110 asthma patients had an ANA titre ≥1:160, compared to 0% of 92 COPD patients (75). A high eosinophil blood count was a negative predictor of ANA in asthma, however, no association with disease severity in asthma was found. However, given the frequencies of both asthma and autoimmune disease it is possible that 10% of the asthma cohort has an underlying autoimmune condition. Mukherjee et al. assessed anti-eosinophil peroxidase (EPX) IgG and ANA/ENA in 51 patients with eosinophilic asthma (EA) compared to 15 healthy controls (76). They found that both anti-EPX IgG and ANA were greater in the sputum of patients with prednisone-dependent eosinophilic asthma and in those with severe asthma and pleiotropic bronchitis compared to those with mild-to-moderate EA or neutrophilic asthma and healthy controls. Where available, serum was tested but the anti-EPX IgG response was absent, suggesting a polyclonal immune response localized to the airways. Furthermore, sputum with high antibody titres resulted in greater induced eosinophil granulation as measured by LDH release and dsDNA in the supernatant. Finally, they found that approximately one third of those with severe asthma had autoantibodies against eosinophilic cellular components in their sputum. Qin et al. assessed both sputum and serum autoantibodies in asthma of varying severities (mild=15, moderate=18, severe=18) versus healthy controls (n=24) (77). Assessment of 10 different autoantibodies (PR3, P0, SSA, SSB, Scl-70, Jo-1, U1-SnRNP, Sm, MPO and thyroid peroxidase (TPO)), found sputum antibodies were more associated with clinical parameters and severity of disease than serum autoantibodies. Sputum anti-Sm, U1-snRNP and TPO autoantibodies were higher in severe asthma than mild asthma or healthy controls although the individual group sizes for these subsets were too small to make any strong conclusions.

These studies suggest that there may be an airway-localised autoimmune phenomenon in a subset of severe (possibly eosinophilic) asthma, and they may correlate with disease activity better than serum antibodies. However, none of this work has yet been replicated in separate or large cohorts and so more research and validation studies are required to determine any clinical significance.

#### Miscellaneous

Other autoantibody studies in asthma include antibodies to collagen V, PPL, and IL-33.

A study assessing anti-collagen V autoantibodies found elevated levels in patients with asthma above that in healthy controls and this correlated with increasing daily corticosteroid dosage but no other clinic-demographic variables (78). Anti-PPL autoantibodies were assessed by Taille et al. after their previous findings of anti-PPL autoantibodies in IPF, and found these antibodies also more frequent in asthma patients compared to healthy controls but the presence of antibodies did not correlate with severity (79). Ji et al. assessed antibodies to 11 cytokines, in 137 patients with asthma in comparison with 96 healthy controls (80). Interestingly, only anti-IL-33 autoantibodies were significantly increased. IL-33 is an alarmin cytokine that can drive Type 2 immune responses. Neutralising monoclonal antibodies to IL-33 have been tested in clinical trials in severe asthma. There was a higher proportion of mild-moderate asthmatic cases (28%) compared to severe (6%) cases that were positive for anti-IL-33 autoantibodies (p=0.0002), suggesting a negative correlation with severity and that these antibodies may be protective. This was further supported in allergic asthma patients, where anti-IL-33 autoantibodies correlated negatively with serum concentrations of a number of Type 2 cytokines (IL-4, IL-13, IL-25, IL-33), IgE, blood eosinophil, lymphocyte and basophils counts and positively correlated with improved lung function (FEV1%). The antibodies were polyclonal IgG and could neutralise IL-33-induced pathogenic responses in an *in vitro* IL-33 neutralisation assay and *in vivo* in a BALBc mice model of rIL-33 induced lung inflammation.

Overall, specific subsets of asthma may be associated with autoantibody presence. Cytokeratin autoantibodies may be positively correlated with disease and there is robust evidence to suggest that anti-IL-33 autoantibodies may play a protective role in disease severity. The latter is a highly unique observation as other autoantibodies in respiratory diseases appear to either be linked to pathogenicity or be bystander biomarkers.

### Sarcoidosis

Sarcoidosis is a multisystem inflammatory disease characterized by non-caseating granulomas in various organs. The pathogenesis is unknown, and an autoimmune mechanism has been hypothesized but remains unconfirmed. Eight studies assessing autoantibodies are reviewed below. Overall, no autoantibodies were found to be different in sarcoidosis patients compared to healthy controls in three studies when assessing for routine diagnostic autoantibodies. These antibodies included anti-CCP which were not found to be elevated in sarcoidosis patients (81–83); and a variety of routine diagnostic autoantibodies (ANA (≥1:100 as positive via IIF), RF, ENA, dsDNA, β2GP1, cardiolipin, TPO, thyroglobulin, ANCA) (83).

#### Vimentin

One group published two studies assessing autoantibodies to mutated citrullinated vimentin (MCV) in sarcoidosis patients. Malkova et al. described a cohort of 93 sarcoidosis patients and compared them to patients with tuberculosis (TB) (n=28) and healthy controls (n=40) (84). Anti-MCV autoantibodies were elevated in 38/93 (40.9%) of sarcoidosis patients and 17/28 (60.7%) of TB patients (p=0.027), and 3/40 (7.5%) cases of healthy controls (compared to both other groups, p<0.0001). Starshinova et al. assessed these 93 sarcoidosis patients and also tested those positive for anti-MCV autoantibodies for CCP antibodies, citrullinated vimentin (anti-Sa antibodies), and non-modified vimentin, and compared them to various non-infectious lung disease controls (n=55) and healthy controls/tuberculosis contacts (n=40) (82). In the 38 sarcoidosis patients and controls with anti-MCV autoantibodies, CCP antibodies (as discussed above) were positive in only one sarcoidosis patient, two patients in the lung disease control group and 1 healthy control. Anti-Sa antibodies were seen in 13 sarcoidosis patients, and 9 lung disease control group patients (healthy controls not discussed). A moderate relationship (r=0.66, p=0.017) was found between the anti-MCV and anti-Sa autoantibody levels in the 13 autoantibody positive patients with pulmonary sarcoidosis although the numbers are too small to be reliable. Overall, this study suggests that anti-MCV and anti-citrullinated vimentin autoantibodies can be seen in sarcoidosis patients, but their clinical significance remains unclear.

#### Miscellaneous

Khassawneh et al. performed a 128-plex protein array to assess for antibodies to 120 autoantigens in sera from 236 sarcoidosis patients (split into 106 pulmonary involvement only (POS) and 120 extrapulmonary involvement (EPS)) compared to 101 healthy controls (85). It is not clear why this panel specifically was selected. The results showed 5 IgG antibodies that were significantly elevated in sarcoidosis compared to healthy controls (antibodies to each of CRP, elastin, heparin, myelin basic protein and vimentin), and 58 elevated IgM antibodies were found in sarcoidosis compared to healthy controls. Stepwise multiple logistic regression analysis on the differentially elevated autoantibodies identified 11 IgM antibodies which can efficiently distinguish sarcoidosis patients from healthy controls. The panel of 11 IgM antibodies included BPI (also frequently observed in CF), fibrinogen IV, hemocyanin, histone H2B, histone H3, matrigel, prothrombin protein, Smith, Smith subunit D, TPO and vimentin. Although the data is promising their panels are not exhaustive and validation of autoantibody specificities is required.

#### Unbiased discovery-based autoantibody screening technologies

Haggmark et al. assessed 345 patients: sarcoidosis without Lofgren’s syndrome (n=140), sarcoidosis with Lofgren’s (n=139), healthy controls (n=49), and allergic asthma (n=17) (86). Using a protein microarray for 3072 protein fragments, they measured autoantibodies in BALf, along with matched serum samples in a subset (sarcoidosis, n=123, healthy controls, n=18) and found four new autoantigen targets. This included zinc finger protein 688 (ZNF688), adenosine diphosphosphate ribosylation factor GTPase (ARFGAP1), mitochondrial ribosomal protein L43 (MRPL43) and nuclear receptor coactivator 2 (NCOA2). Inhibition assays confirmed reactivity to these antigens. In paired serum samples, correlations of signal intensities showed high concordance with BALf autoantibodies to these targets (rho=0.81).

Baerlecken et al. searched for novel autoantigens in sarcoidosis patients via a 28,000 recombinant protein planar array albeit with a very small discovery sample size (resulting in a possible false negatives bias) (87). They found one autoantigen that was common between three sarcoidosis screening patients, negative elongation factor E (NELF-E). NELF-E is a widely expressed protein and part of the NELF complex, which is a multi-subunit complex which cooperates with DRB Sensitivity Inducing Factor (DSIF) to express RNA polymerase II elongation. They then recruited 82 sarcoidosis patients and compared them to 259 controls. Anti-NELF-E IgG was detected in 29/82 (35%) sarcoidosis patients, compared to 3/46 (6.5%) of febrile infectious diseases group, 4/36 (11%) patients with SLE, 3/64 (4.7%) in PET controls, 8/100 (8%) blood donors, and 0/13 healthy controls. Clinical associations found that patients with detectable antibodies showed more disease involvement of the lung parenchyma compared to seronegative patients on x-ray. The median antibody titre was also higher with lung parenchymal involvement.

Peng et al. built on their previous study (88) in which two novel neoantigens were discovered using a custom-made T7 phage display library of peptides derived from sarcoidosis cDNA isolated from BALf and peripheral blood cells (89). The two neoantigens were cofilin-μ, a peptide showing partial homology to cofilin-1, a regulator of actin filament dynamics, and chain A, showing partial homology to methionine aminopeptidase 1 (MetAP1), which is involved in protein processing. These were then validated in a cohort of 186 sarcoidosis patients, 100 healthy controls, 70 patients with pulmonary TB, and 30 lung cancer patients. For both neoantigens, significant differences in IgG autoantibodies were found in the sarcoidosis cohorts versus healthy controls, TB and lung cancer subjects. In addition, ROC analysis showed high sensitivity, specificity, accuracy, NPV, and PPV for both antibodies, suggesting that ELISA-based detection of these IgG autoantibodies could have potential to distinguish sarcoidosis from other groups including TB.

### Autoimmune pulmonary alveolar proteinosis

aPAP has a well described association with autoantibodies and was not included in the initial search strategy of this review. However, two studies assessing ILD included anti-GM-CSF antibodies. Both studies compared aPAP patients to a variety of other respiratory diseases and healthy controls (90, 91). Both found elevated serum anti-GM-CSF antibodies in aPAP to be markedly increased compared to other respiratory diseases such as sarcoidosis, pneumoconiosis, and hypersensitivity pneumonitis.

## Discussion

In this scoping review we performed a systematic literature search assessing 79 studies that report on the presence of autoantibodies in primary non-infectious chronic respiratory diseases.

For IPF, most commonly, presence of routine autoantibodies were assessed which can be anticipated given pulmonary fibrosis is a common and well described complication of connective tissue disease. However, it is important to note our manuscript only incorporated studies in which IPF was not considered secondary to an underlying SARD. BPIFB1 appears to be linked to T-cell immune responses and the associated autoantibodies may be represent bystanders as passive transfer did not induce ILD *in vivo.* COPD studies were heterogenous, with a large variety of autoantigens being assessed with unbiased approaches but CK18 appeared promising. In Cystic Fibrosis, anti-BPI autoantibodies were found to be associated with anti-pseudomonal serology as well as chronic *P. aeruginosa* colonization. In non-CF bronchiectasis, studies focused on CCP, RF and ANCA, likely reflecting systemic diseases that are often complicated by bronchiectasis as a secondary comorbidity, although this represents only a small fraction of total bronchiectasis cases. In asthma, both routine autoimmune assays and anti-cytokeratin autoantibodies were assessed, with the novel finding of a potentially protective autoantibody neutralizing IL-33. Sarcoidosis is not yet linked to a particular autoantigen although this review identifies several emerging candidates that now require validation using independent and larger cohorts. For silicosis, no eligible studies evaluating autoantibody profiles were identified despite inclusion in the search strategy and its association with autoimmunity. This absence may reflect a true gap in the literature but could also be influenced by differences in terminology, or limited publication of mechanistic immunological studies in this population. Although aPAP was not specifically included in the search strategy, given anti-GM-CSF antibodies are well established clinically we decided to include these studies for comprehensiveness (90, 91).

Although several autoantibody–disease associations were consistently observed across cohorts, providing emerging clinical evidence, a key limitation of this review was the targeted approach implemented by the majority of the included studies. There is a fundamental difference in interpreting studies using a targeted approach versus an unbiased global or semi-global approach. There are currently very few studies employing the latter for respiratory diseases. A targeted approach is limited to candidates from a preconceived hypothesis typically based on markers associated with other systemic autoimmune diseases and common autoantigens. While this approach can include large sample numbers and frequently assesses validated and/or robust clinical tests, it limits discovery. In contrast, unbiased screens utilize a large panel of antigens without bias towards specific targets to detect autoantibodies across the whole or large proportions of the human proteome. While this approach is more resource intensive for large sample numbers and lacks the validity of established clinical diagnostic tests, positive hits can still be validated with more standard approaches (such as ELISAs) once targets are identified. A classic unbiased screen technology is SEREX, where tissue-derived antigens are assessed by recombinant expression cloning through a cDNA phage library also amplified in *E coli* (92). A more recent and powerful screening technology is Phage Immunoprecipitation Sequencing (PhIP-seq) which, despite limitations in complex protein folding, enables high-throughput screening of >100,000 short peptides using phage display to cover the whole human proteome (93). Protein microarrays are semi-untargeted; they utilise larger protein sequences but are low throughput and less efficient. They are limited to a smaller number of autoantigens as well as a smaller number of serum/antibody samples. These arrays also often lack tertiary structure of the proteins. REAP enables multiplexed detection of extracellular (membrane and secreted) autoantigens using a yeast library but is limited with regards to intracellular autoantigen detection and while using many antigen targets to probe serum/antibodies against, REAP still only covers <20% of the human proteome (94). Selection of one or more of the above technologies follows consideration of sample number (disease heterogeneity), throughput, cost, proportion of the proteome to assess, prioritization of peptide sequence-specific autoantibodies or tertiary protein structures and/or PTMs such as phosphorylation and glycosylation of protein domains. No current technology is optimal for all these considerations.

After discovery and validation, in future research the significance of the detected autoantibodies should be determined, which includes several aspects:

1. The functional relevance i.e. whether antibodies are pathogenic, epiphenomena, or protective against disease. Some studies propose significant correlation of autoantigens, such as BPIFB1 in ILD, but the associated autoantibodies have not been demonstrated to be pathogenic and validation of these findings would be important (26).
2. Replication of associations with autoantibodies in sufficiently large validation cohorts. Validation cohorts should include patients with other respiratory or autoimmune diseases. For most autoantibodies and diseases in this review, with some notable exceptions, findings have not been validated. Validation is critical for reproducibility and to ensure that associations of autoantibodies with a disease biomarker are not over-interpreted for their relevance. Comparison to healthy controls may infer a magnitude of difference which is not true when compared with patients with other unrelated respiratory diseases.
3. Large control groups are essential because the background of autoantibody production to diverse antigens occurs normally in the general healthy population. Therefore, a comparative study must include a control sample large enough to avoid this type I error.
4. Unbiased approaches using new technologies are required to identify novel autoantibodies. This approach is essential to progress this field, both to assess new biomarkers but also to understand how autoantibodies may contribute to pathogenesis. This review clearly demonstrates that most studies have instead assessed a small number of specific antigens selected either from hypothesis-generated targets or from commonly available clinical tests used to assess systemic autoimmune diseases.

Although many studies report novel autoantibody associations, replication in independent cohorts and functional validation remain limited. Consequently, most reported autoantibodies are supported primarily by associative evidence rather than demonstrated mechanistic involvement in disease pathogenesis, and their clinical relevance remains uncertain and should be interpreted with caution.

It is possible that specific patient subgroups within a disease cohort produce functional or neutralizing autoantibodies unique to that subset. Identification of these subgroups however, is more likely in large cohorts and underlines the need for substantial sized studies. Information about these subsets can be highly relevant as these autoantibodies may identify clinically distinct subsets (endotypes) and help improve prognostication, disease monitoring, prediction and/or treatment with immune modulation. The pathway from autoantibody discovery and functional validation to translation into clinical practice requires significant resources but with discovery and development of targeted therapies patient outcomes can be improved.

This study has several limitations. The review scope was refined *post hoc* to focus on non-infectious respiratory diseases after initial data extraction. Although this improved conceptual clarity and focus, it may have led to the exclusion of studies that could provide complementary insights into overlapping disease mechanisms with infectious disease. Similarly, because the search strategy was developed prior to this refinement, it may not have exhaustively captured every potential relevant study within the non-infectious domain. Moreover, some of the included diseases, such as cystic fibrosis, bronchiectasis or COPD, are frequently associated with chronic infection or microbial colonisation, and therefore some reported autoantibodies may reflect infection-driven immune responses rather than disease-specific autoimmune mechanisms, complicating interpretation of their pathogenic and biomarker significance.

Three studies were added prior to final synthesis that were missed in the initial search that provided novel data. Some of the missed articles may have been due to the broader search strategy, restrictive case-control and cohort study filters or erroneous Medical Subject Heading (MeSH) classifications associated with the deposited publication. Whilst the database search was comprehensive, misleading titles may have led to inadvertent exclusion during screening. For example, the lack of studies associated with autoantibodies in silicosis should be interpreted cautiously as an absence of evidence rather than definitive evidence of absence.

As expected, there was substantial methodological heterogeneity amongst the included studies. Differences in study design, sample size, patient selection, control groups and autoantibody detection methods makes direct comparison across studies challenging. As a scoping review, formal risk of bias assessment was not performed. Moreover, minimal sample size thresholds were applied as pragmatic screening criteria to balance inclusivity with exclusion of very small exploratory case reports and case series whilst maintaining sensitivity to potentially relevant emerging autoantibody signals. Therefore the findings should be interpreted as summarising existing evidence rather than evaluation of quality or certainty.

A further conceptual limitation relates to the distinction between primary respiratory disease and interstitial lung disease associated with systemic autoimmune rheumatic diseases (SARD). Interpretation of autoantibodies in chronic respiratory disease is complicated by uncertainty regarding their biological origin. The review focused on primary respiratory diseases to attempt to better delineate disease mechanisms intrinsic to pulmonary pathology. Studies of connective tissue-related secondary respiratory disease were excluded as they are more likely to reflect systemic rather than pulmonary immune-mechanisms. Whilst this approach increased conceptual homogeneity, classification overlap between primary ILD for example versus ILD secondary to SARD represents an inherent limitation. Some excluded studies of CTD-related diseases may still have been mechanistically relevant, as they describe similar or low-titre autoantibodies. Conversely, several included studies classified as non-CTD related respiratory disease may have incorporated participants with early or subclinical systemic autoimmune disease. Moreover, depending on disease context, reported autoantibodies may reflect lung-intrinsic immune dysregulation, occult or pre-clinical systemic autoimmunity or other mechanisms that remain poorly understood. This boundary ambiguity is an inherent limitation of the available literature and contributes to heterogeneity in reported findings, though also reflects real-world diagnostic ambiguity.

Despite these limitations, to our knowledge this represents the most comprehensive summary available to date for autoantibodies in primary non-infectious chronic respiratory diseases. The findings suggest that many chronic respiratory diseases may be heterogenous in nature and disease pathogenesis may in some cases be driven by autoimmune mechanisms and endotypes marked by autoantibodies.

## Conclusion

In this scoping review, we summarise the broad body of literature evaluating autoantibody responses in a variety of chronic non-infectious respiratory diseases. Autoantibodies are frequently reported across these conditions and may identify biologically distinct subgroups. However, evidence for direct pathogenic causality remains limited to a small number of antibody–disease contexts where functional validation has been demonstrated. Future research with unbiased studies followed by rigorous functional characterisation is required to determine biological origin and clinical significance of these autoantibody responses.

## Data Availability

The original contributions presented in the study are included in the article/**Supplementary Material**. Further inquiries can be directed to the corresponding author.
