# Supplementary material for: Autoantibodies in chronic non-infectious respiratory diseases: a scoping review
Source: Front Immunol. 2026 Jul 28;17:1809141. doi: 10.3389/fimmu.2026.1809141 (PMC13457196; doi:10.3389/fimmu.2026.1809141)
Supplement: Supplementary file 1 [file Table1.docx]

**Embase <until 2024 September 13>**

1	autoantibody/

2	autoantibod*.mp. [mp=title, abstract, heading word, drug trade name, original title, device manufacturer, drug manufacturer, device trade name, keyword heading word, floating subheading word, candidate term word]

3	"auto antibod*".mp. [mp=title, abstract, heading word, drug trade name, original title, device manufacturer, drug manufacturer, device trade name, keyword heading word, floating subheading word, candidate term word]

4	"self reactive".mp. [mp=title, abstract, heading word, drug trade name, original title, device manufacturer, drug manufacturer, device trade name, keyword heading word, floating subheading word, candidate term word]

5	"self antigen".mp. [mp=title, abstract, heading word, drug trade name, original title, device manufacturer, drug manufacturer, device trade name, keyword heading word, floating subheading word, candidate term word]

6	autoantigens.mp. [mp=title, abstract, heading word, drug trade name, original title, device manufacturer, drug manufacturer, device trade name, keyword heading word, floating subheading word, candidate term word]

7	1 or 2 or 3 or 4 or 5 or 6

8	exp bronchiectasis/

9	bronchiectasis.ti,ab,kf.

10	8 or 9

11	exp lung fibrosis/

12	("pulmonary fibrosis" or "interstitial lung disease").ti,ab,kf.

13	11 or 12

14	cystic fibrosis/

15	"cystic fibrosis".ti,ab,kf.

16	14 or 15

17	coronavirus disease 2019/

18	("covid 19" or "sars cov 2").ti,ab,kf.

19	17 or 18

20	exp respiratory tract infection/

21	("pneumonia" or "respiratory tract infection").ti,ab,kf.

22	20 or 21

23	exp pneumoconiosis/

24	"pneumoconiosis".ti,ab,kf.

25	23 or 24

26	exp sarcoidosis/

27	sarcoidosis.ti,ab,kf.

28	26 or 27

29	exp chronic obstructive lung disease/

30	("emphysema" or "chronic obstructive pulmonary disease").ti,ab,kf.

31	29 or 30

32	exp asthma/

33	asthma.ti,ab,kf.

34	32 or 33

35	10 or 13 or 16 or 19 or 22 or 25 or 28 or 31 or 34

36	7 and 35

37	exp animal/ not human/

38	36 not 37

39	exp cohort analysis/

40	exp longitudinal study/

41	exp prospective study/

42	exp follow up/

43	cohort$.tw.

44	exp case control study/

45	(case$ and control$).tw.

46	39 or 40 or 41 or 42 or 43 or 44 or 45

47	38 and 46

**Ovid MEDLINE® <until 2024 September 13>**

1	autoantibodies/ or anti-citrullinated protein antibodies/ or antibodies, antineutrophil cytoplasmic/ or antibodies, antinuclear/ or rheumatoid factor/

2	autoantibod*.mp.

3	"auto antibod*".mp.

4	"self reactive".mp.

5	"self antigen".mp.

6	autoantigens.mp.

7	1 or 2 or 3 or 4 or 5 or 6

8	bronchiectasis/ or kartagener syndrome/

9	bronchiectasis.ti,ab,kf.

10	8 or 9

11	pulmonary fibrosis/ or idiopathic pulmonary fibrosis/ or sarcoidosis, pulmonary/ or granulomatosis with polyangiitis/

12	("pulmonary fibrosis" or "interstitial lung disease").ti,ab,kf.

13	11 or 12

14	Cystic Fibrosis/

15	"cystic fibrosis".ti,ab,kf.

16	14 or 15

17	COVID-19/

18	("covid 19" or "sars cov 2").ti,ab,kf.

19	17 or 18

20	exp respiratory tract infection/

21	("pneumonia" or "respiratory tract infection").ti,ab,kf.

22	20 or 21

23	exp Pneumoconiosis/

24	"pneumoconiosis".ti,ab,kf.

25	23 or 24

26	exp Sarcoidosis/

27	"sarcoidosis".ti,ab,kf.

28	26 or 27

29	exp Pulmonary Disease, Chronic Obstructive/

30	("emphysema" or "chronic obstructive pulmonary disease").ti,ab,kf.

31	29 or 30

32	exp Asthma/

33	asthma.ti,ab,kf.

34	32 or 33

35	10 or 13 or 16 or 19 or 22 or 25 or 28 or 31 or 34

36	7 and 35

37	exp animals/ not humans.sh.

38	36 not 37

39	exp cohort studies/

40	cohort$.tw.

41	exp case-control studies/

42	(case$ and control$).tw.

43	39 or 40 or 41 or 42

44	38 and 43
