# Supplementary material for: Autoantibodies in chronic non-infectious respiratory diseases: a scoping review
Source: Front Immunol. 2026 Jul 28;17:1809141. doi: 10.3389/fimmu.2026.1809141 (PMC13457196; doi:10.3389/fimmu.2026.1809141)
Supplement: Supplementary file 2 [file Table2.docx]

**IPF: Routine autoantibodies in clinical assessment**

| **Study author & year** | **Primary country** | **Cases/ controls (n)** | **Severity of cases/details of controls (if relevant)** | **Antigens studied** | **Assay used** | **Functional assays** |
| --- | --- | --- | --- | --- | --- | --- |
| Lee 2013 | USA | 67/52 | Cases: IPF (67)  Controls: HC age 50-80 years (52).  Other, not included: UCTD-ILD (22) | ANA  RF  CCP  ENA  dsDNA  Vasculitis | IIF (ANA ≥1:320)  Nephelometry (RF)  BioPlex (all others) | N/A |
| Moua 2014 | USA | 389/0 | Cases: biopsy-confirmed IPF | ANA  ENA  CCP  RF  dsDNA | IIF (ANA, >1:80), ELISA<1 | N/A |
| Goobie 2019 | Canada | 61/0 | Cases: IPF pts without CTD signs/symptoms and had serologic testing (61) | ANA  ENA | IIF (ANA, pos ≥1:320)  ALBIA (ENA) | N/A |
| Ghang 2019 | South Korea | 512/0 | Cases: ab positive (138), ab negative (374) | ANA  ENA  ANCA  MPO  PR3  RF  CCP  dsDNA | IIF (ANA pos ≥1:40)  Others not specified | N/A |
| Wang 2021 | China | 222/0 | Cases: IPF (222) | ANA  ANCA  RF | N/A | N/A |
| Kirgou 2022 | Greece | 102/0 | Cases: IPF (102) | ANA  ENA  RF  ANCA  CCP  dsDNA (minimal detail) | IIF (ANA, pos >1:80, ANCA)  ELISA  Blot assays | N/A |
| Song 2021 | South Korea | 234/0 | Cases: IPF pts never treated with pirfenidone (92), pts who received pirfenidone for >6 months (142) | ANA  ENA  ANCA  RF  CCP  dsDNA  MPO PR3 | IIF (ANA, ANCA, ≥1:320 pos)  Others not specified | N/A |
| Izhakian 2024 | Israel | 161/0 | Cases: IPF pts | ANA | IIF (ANA, high titers≥1:160, low titers <1:160) | N/A |
| Hozumi 2016 | Japan | 16/94 | Cases: PR3-ANCA+ IIP (16), with 3 being PR3-ANCA IPF  Controls: ANCA-negative IPF (94) | ANCA  PR3 | ELISA | N/A |
| Solomon 2020 | USA | 189/257 | Cases: IPF from primary cohort (88), validation cohort (101)  Controls: general population (160), RA-UIP (40), HP (46), Lung Tissue Research Consortium (11) | CCP IgA/IgG | ELISA | N/A |

**IPF: Periplakin**

| **Study author & year** | **Primary country** | **Cases/ controls (n)** | **Severity of cases/details of controls (if relevant)** | **Antigens studied** | **Assay used** | **Functional assays** |
| --- | --- | --- | --- | --- | --- | --- |
| Taille 2011 | France | 40/60 | Cases: IPF (40) who had negative ANA >1:160 and RF  Controls: HC (40), CTD-lung fibrosis (10), COPD (10) | Periplakin | Immunoblot with placental extract | PPL inhibition experiment  Epithelial cell migration using alveolar epithelial wound repair assay |
| Koether 2023 | France | 46/36 | Cases: IPF (46)  Controls: HC (36) | Periplakin  HSP70  Vimentin | Western blot | Human lung sample immunohistochemistry and IF for antigen  Mouse lung fibrosis model |

**IPF: Vimentin**

| **Study author & year** | **Primary country** | **Cases/ controls (n)** | **Severity of cases/details of controls (if relevant)** | **Antigens studied** | **Assay used** | **Functional assays** |
| --- | --- | --- | --- | --- | --- | --- |
| Li 2017 | USA | 172/156 | Cases: Discovery cohort, IPF from Uni of Alabama (102), Replication cohort, IPF from Uni of Pittsburgh (70)  Controls: non-IPF (50, 60), COPD (46, discovery cohort only) | Vimentin | ELISA | N/A |
| Le Guen 2024 | France | 86/0 | Cases: IPF (86) | Vimentin | ELISA | N/A |

**IPF: Miscellaneous**

| **Study author & year** | **Primary country** | **Cases/ controls (n)** | **Severity of cases/details of controls (if relevant)** | **Antigens studied** | **Assay used** | **Functional assays** |
| --- | --- | --- | --- | --- | --- | --- |
| Kahloon 2013 | USA | 122/111 | Cases: IPF (122)  Controls: normal volunteers (60), subjects with ILD other than IPF (51) | HSP70  GRP78 | Immunoprecipitation of cell lysate proteins from pooled plasma, mass spec  Immunoblot/ELISA for confirmation | Monocyte stimulation assay with assessment of surface CD69 and production of IL8 in supernatant |
| Mills 2019 | UK | 107/85 | Cases: IPF progressors (30), IPF non-progressors (52), IPF without follow up (25)  Controls: non-IPF ILD (66, including 13 HP, 11 NSIP, 42 unclassifiable), HC (19) | HSP72 | ELISA | Monocyte cell culture and expression of CXCL8 with inhibition assay |
| Shum 2013 | See non-IPF-ILD |  |  |  |  |  |
| Beltramo 2018 | France | 138/0 | Cases: IPF with ACPA+ (138) | Parietal cell | IIF (≥1:80 pos) | N/A |
| Philippot 2023 | France | 247/36775 | Cases: IPF (247)  Controls: general population | IFN-α  IFN-ω  IFN-β | Gyros (IFNα, IFNω) | Luciferase reporter assay (IFNα, IFNω, IFNβ) |
| Arai 2022 | Japan | 71/0 | Cases: IPF (71) | Myxovirus resistance protein 1 (MX1) IgG/A/M | ELISA | N/A |

**IPF: Unbiased**

| **Study author & year** | **Primary country** | **Cases/ controls (n)** | **Severity of cases/details of controls (if relevant)** | **Antigens studied** | **Assay used** | **Functional assays** |
| --- | --- | --- | --- | --- | --- | --- |
| Ujike-Hikichi 2023 | Japan | 64/61 | Cases: ILD (IPF 43, fNSIP 21)  Controls: HC (61) | UBE2T | Protein microarray (9483 antigens)  Confirmation using ELISA | N/A |

**IPF: Manually added unbiased**

| **Study author & year** | **Primary country** | **Cases/ controls (n)** | **Severity of cases/details of controls (if relevant)** | **Antigens studied** | **Assay used** | **Functional assays** |
| --- | --- | --- | --- | --- | --- | --- |
| Kurosu 2008 | Japan | 45/180 | Cases: IPF (45), made up of acute exacerbation (15), stable (30)  Controls: HC (40), interstitial pneumonia related to collagen vascular diseases (90; PM/DM=20, SjS=20, SSC=20, RA=20, SLE=10), sarcoidosis (30), chronic eosinophilic pneumonia (10), hypersensitivity pneumonitis (10) | Annexin I | SEREX  ELISA  Western blot  Lymph node in vitro assessment  Done in BAL and sera | N/A |

**Non-IPF ILD**

| **Study author & year** | **Primary country** | **Cases/ controls (n)** | **Severity of cases/details of controls (if relevant)** | **Antigens studied** | **Assay used** | **Functional assays** |
| --- | --- | --- | --- | --- | --- | --- |
| Bermea 2019 | USA | 520/0 | Cases: IIP (including IPF, NSIP and biopsy proven organizing pneumonia), without CTD | RNA-binding protein (RBP), including SSA, SSB, Sm, RNP | ELISA | N/A |
| Morita 2020 | Japan | 243/0 | Cases: IIP without CTD (243). Does not include those with CTD (42) | ANA (>1:40)  Myositis/Ro52 | IIF (ANA)  Myositis line blot (Ro52) | N/A |
| Correia 2019 | USA | 2030/0 | Cases: confirmed RA (453), unconfirmed RA (1577) | CCP  RF (not a focus) | ELISA | N/A |
| Zheng 2022 | Canada | 1751/0 | Cases: 1570 non-CTD ILD (IPF, hypersensitivity pneumonitis, IPAF or unclassifiable ILD), RA-ILD (181). | RF  CCP | N/A | N/A |
| Shum 2013 | USA, Sweden | 156/150 | Initially  Cases: APS1 pts (104)  Controls: HC (126), COPD (51), asthma (51)  Cases: ILD (108), made up of IPF (58), NSIP (17), bronchiolitis (4), COP (4), unclassifiable (25)  Also CTD-ILD (48), made up of undifferentiated CTD (17), scleroderma (16), RA (7), dermatomyositis (5), MCTD (3).  Controls:  Other, without evidence of ILD (150), including HC (46), T1DM (42), T2DM (35), non-ILD lung disease (27) | BPIFB1  IFN-α | Radioligand binding assay  IIF staining of frozen lung sections with competition assay to confirm specificity of bronchiolar staining pattern | Mice models with immunisation of mice with anti-BPIFBI (negative), T cells (positive) or Aire-/- thymi (positive) |

**Non-IPF ILD: Unbiased**

| **Study author & year** | **Primary country** | **Cases/ controls (n)** | **Severity of cases/details of controls (if relevant)** | **Antigens studied** | **Assay used** | **Functional assays** |
| --- | --- | --- | --- | --- | --- | --- |
| Upadhyay 2024 | USA | 398/184 | Cases: ILD (398), made up of CTD-ILD (80), hypersensitivity pneumonitis (70), IPF (102), interstitial lung abnormality (10). Other (81), unclassifiable (28), unknown CTD-ILD (27)  Controls: HC blood bank (138), non-ILD controls (46) | Unbiased  CDHR5 | PhIP-Seq  RLBA, RNA sequencing and immunohistochemistry | N/A |

**IIP: Manually added unbiased**

| **Study author & year** | **Primary country** | **Cases/ controls (n)** | **Severity of cases/details of controls (if relevant)** | **Antigens studied** | **Assay used** | **Functional assays** |
| --- | --- | --- | --- | --- | --- | --- |
| Hamano 2017 | Japan | Screen: 18/10  Confirmation-  Cohort 1: 114/30  Cohort 2:  152/0 | Cases: IPF (10), iNSIP (8), aPAP (10), sarcoidosis (10)  Controls: HC (10)  Confirmation cohorts:  Chronic fibrosing IIPs (114), including IPF (19), non-IPF (95), and HC (30)  Chronic fibrosing IIPs (155), including IPF (71), non-IPF (84) | Unbiased  MX1 (IgG/A/M) | Protein microarray  Immunoprecipitation  ELISA | N/A |

**Cystic fibrosis: BPI**

| **Study author & year** | **Primary country** | **Cases/ controls (n)** | **Severity of cases/details of controls (if relevant)** | **Antigens studied** | **Assay used** | **Functional assays** |
| --- | --- | --- | --- | --- | --- | --- |
| Lindberg 2015 | Sweden | 117/0 | Cases: non-transplanted CF (117) | BPI IgA  Pseudomonas IgG | ELISA (BPI)  EIA (pseudomonas E15 detecting ab against ALP, ExoA, ELA) | N/A |
| Skopelja 2016 | USA | 38/50 | Cases: adult CF (38)  Controls: RA (50) | nBPI  CCP  RF  Carbamylated proteins | ELISA | NET induction and immunocytochemistry |
| Theprungsirikul 2020 | USA | 131/0 | Cases: adult CF (23 serum, 18 BAL), adult and pediatric (32 and 24 serum), pediatric cohort (20 BAL), pediatric CF (14 BAL) | BPI (IgG in serum, IgG/A in BAL)  Pseudomonas aeruginosa (IgG in serum, IgG/A in BAL) | ELISA  Immunoblot | N/A |
| Iwuji 2023 | USA | 47/5 | Cases: CF (47)  Controls: HC (5) | BPI | ELISA | N/A |

**Cystic fibrosis: Miscellaneous**

| **Study author & year** | **Primary country** | **Cases/ controls (n)** | **Severity of cases/details of controls (if relevant)** | **Antigens studied** | **Assay used** | **Functional assays** |
| --- | --- | --- | --- | --- | --- | --- |
| Lachenal 2009 | France | 144/0 (92 blood donors for ASCA /ANCA only) | Cases: adult CF pts (144) | Actin  ANA (>1:80)  ASCA IgA  ENA  ANCA (and their specificities)  CCP  RF  dsDNA  Endomysial  GAD  IA2  Thyroglobulin  Tissue  TPO  TTG | Various; includes ELISA, IIF and RIA | N/A |
| Greene 2010  Also discussed in COPD | Ireland | 91/33 | Cases: COPD (31), Z-A1AT def (20), CF (40)  Controls: HC (33) | Elastin  N-Ac-PGP (proline glycine proline)  ANA | ELISA (elastin, N-Ac-PGP)  IIF (ANA, pos ≥1:80) | N/A |
| Hirche 2011 | Germany | 119/88 | Cases: stable CF, children (70), adults (49)  Controls: crohn’s disease (34), ulcerative colitis (11), RA (22), HC (16) | Anti-glycan antibodies e.g.:  ASCA IgG  ALCA (laminaribioside carbohydrate) IgG  ACCA (chitobioside carbohydrate) IgA, AMCA (mannobioside carbohydrate) IgG | ELISA | N/A |
| Janssen 2015  Also discussed in bronchiectasis | Netherlands | 235/122 | Cases: CF patients (41), non-CF patients with bronchiectasis (80), RA with periodontal disease (114)  Controls: HC (36), RA without lung disease with known periodontal status (86) | CCP (IgG, IgA)  Carbamylated proteins (IgG, IgA)  Citrullinated proteins (IgG, IgA)  RF (IgM, IgA) | ELISA | N/A |
| Yadav 2020 | USA | 168/57 | Cases: 3 cohorts; CF adults providing sputum only (70), CF adults providing serum (32), CF children and adults who gave serum (66)  Controls: HC (27), RA (20), SLE (10) | dsDNA IgA, IgG | ELISA | N/A |
| Linnemann 2022 | USA | 104/0 | Cases: paediatric (44) or adult (60) CF without lung transplant (104)  Controls: paediatric (n=?), adult HC (5) | PAD4 IgG, IgA, IgM  dsDNA IgA | ELISA | N/A |

**COPD: Routine autoantibodies in clinical assessment**

| **Study author & year** | **Primary country** | **Cases/ controls (n)** | **Severity of cases/details of controls (if relevant)** | **Antigens studied** | **Assay used** | **Functional assays** |
| --- | --- | --- | --- | --- | --- | --- |
| Bonarius 2011 | Netherlands | 124/108 | Cases: COPD without smoking history (30), COPD with smoking history (94)  Controls: normal lung function without smoking history (37), and with smoking history (71) | ANA  ANCA  Tissues (mitochondrial, smooth muscle, parietal, LKM) | IIF (ANA pos ≥1:80)  ELISA (ANCA)  IIF (tissues) | N/A |
| Nunez 2011 | Spain | 328/67 | Cases: COPD at first hospital presentation (328)  Controls: healthy smokers (31), or nonsmokers (36) | ANA  ENA  dsDNA  Tissues | IIF (ANA, dsDNA with crithidia, tissues)  ELISA (ENA) | N/A |
| Liang 2019 | China | 102/18 | Cases: stable COPD (102)  Controls: non-smoking healthy controls (18) | ENA  MPO  PR3  TPO | Multiplex bead array (Luminex) | N/A |
| Liang 2020 | China | 102/79 | Cases: discovery cohort of stable COPD (47), validation cohort of stable COPD (55)  Controls: non-COPD controls (59), CTD-ILD (20 | ENA  MPO  PR3  TPO | Multiplex bead array (Luminex) | N/A |

**COPD: BPI**

| **Study author & year** | **Primary country** | **Cases/ controls (n)** | **Severity of cases/details of controls (if relevant)** | **Antigens studied** | **Assay used** | **Functional assays** |
| --- | --- | --- | --- | --- | --- | --- |
| Tian 2018 | China | 126/0 | Cases: chronic COPD and pseudomonas aeruginosa colonization (126) | BPI | ELISA | N/A |
| Tian 2019 | China | 216/344 | Cases: COPD with pseudomonas colonization (216)  Controls: pseudomonas infection but no COPD (244), HC (100) | BPI | ELISA | N/A |

**COPD: Miscellaneous**

| **Study author & year** | **Primary country** | **Cases/ controls (n)** | **Severity of cases/details of controls (if relevant)** | **Antigens studied** | **Assay used** | **Functional assays** |
| --- | --- | --- | --- | --- | --- | --- |
| Cherneva 2012 | Bulgaria | 63/100 | Cases: mild COPD (20), severe COPD (48)  Controls: healthy smokers (52), other acute respiratory tract infection (48) | αB-crystalline (HSPB5) | ELISA | N/A |
| Greene 2010  Also discussed in CF | Ireland | 91/33 | Cases: COPD (31), Z-A1AT def (20), CF (40)  Controls: HC (33) | Elastin  N-Ac-PGP  ANA | ELISA (elastin, N-Ac-PGP)  IIF (ANA, pos ≥1:80) | N/A |
| Bon 2014 | USA | 265/27 | Cases: smokers with COPD (209), smokers without COPD (56). Of 209 COPD patients, 167 had airflow obstruction, 42 had normal spirometry but abnormal lungs  Controls: non-smoker HC (27) | Glucose regulated protein 78 (GRP78) | Immunoblot  IFA on lung specimens for GRP78 | Macrophage stimulation assay |
| Xiong 2017 | China | 228/136 | Cases: COPD (228), with severity as follows: GOLD I (4), GOLD II (41), GOLD III (87), GOLD IV (96)  Controls: HC (136) | CK18  CK19  (IgG/A/M) | ELISA  Lung histology staining of mice, IIF and WB | N/A |
| Shindi 2017 | UK | 54/10 | Cases: COPD (54)  Controls: no COPD but >10 pack year smoking history (10) | Microbial antigens, various cytoplasmic and intracellular antigens, collagens and keratins (IgG/M) | Protein microarray (n=33) | N/A |
| Cass 2021 | Multiple across Europe and North America and China | 141/83 | Two groups:  Europe/North America:  Cases: COPD FEV1>50% (47), COPD FEV1<50% (10)  Controls: asymptomatic smokers (30), HC (28)  China:  Cases: COPD FEV1>50% (44)  Controls: asymptomatic smokers (25) | Multiple (IgG/M) | Multiplex bead array (IgG and IgM ab; 124 antigens) | N/A |

**COPD: Unbiased**

| **Study author & year** | **Primary country** | **Cases/ controls (n)** | **Severity of cases/details of controls (if relevant)** | **Antigens studied** | **Assay used** | **Functional assays** |
| --- | --- | --- | --- | --- | --- | --- |
| Ma 2020 | China | 144/225 | Cases: COPD (144), including acute exacerbation (92), stable (52)  Controls: asthma (101), HC (124) | Unbiased/multiple (19000 proteins)    Lactoferrin | HuProt (unbiased microarray, n=19000)  Immunoblot (lactoferrin IgG) | N/A |

**Bronchiectasis**

| **Study author & year** | **Primary country** | **Cases/ controls (n)** | **Severity of cases/details of controls (if relevant)** | **Antigens studied** | **Assay used** | **Functional assays** |
| --- | --- | --- | --- | --- | --- | --- |
| Soto-Cardenas 2010 | Spain | 41/37 | Cases: primary Sjogrens’ syndrome with bronchiectasis (41)  Controls: Sjogren’s syndrome without bronchiectasis (37)  Bronchiectasis patients without systemic sclerosis (77), antibody data not available | ANA  RF  Smooth muscle  Parietal cell  AMA  aPLs  SSA/SSB | N/A | N/A |
| Neel 2018 | France | 58/0 | Cases: MPA (30), GPA (28), exclusion criteria: EGPA, negative ANCA, non-diagnostic HRCT | ANCA  MPO  PR3 | IIF (ANCA)  EliA Phadia immunoassay (PR3/MPO) | N/A |
| Ren 2022 | China | 48/164 | Cases: bronchiectasis with AAV (48)  Controls: AAV without bronchiectasis (164) | ANA  ANCA  RF | IIF (ANA, ANCA) | N/A |
| Lhote 2020 | France | 61/0 | Cases: adults, MPA (27), GPA (27), EGPA (7) | ANCA  MPO  PR3 | IIF (ANCA)  ELISA | N/A |
| Quirke 2015 | UK | 177/216 | Cases: bronchiectasis alone (122), bronchiectasis and RA (50)  Controls: RA without lung disease (50), asthma (87), healthy control (79) | CCP  RF IgM  Citrullinated proteins (cit α-enolase peptide/CEP1, cit-vimentin, cit-fibrinogen and their argining containing control peptides)  Inhibition/competition assay with CEP1 and REP1 | Automated analyser (RF)  ELISA  (CCP, arginine containing peptides, citrullinated peptides) | N/A |
| Janssen 2015  Also discussed in CF | Netherlands | 235/122 | Cases: CF patients (41), non-CF patients with bronchiectasis (80), RA with periodontal disease (114)  Controls: HC (36), RA without lung disease with known periodontal status (86) | CCP (IgG, IgA)  Carbamylated proteins (IgG, IgA)  Citrullinated proteins (IgG, IgA)  RF (IgM, IgA) | ELISA | N/A |
| Clarke 2017 | UK | 174/216 | Cases: Bronchiectasis (122), bronchiectasis with RA (BRRA, 52)  Controls: HC (79), asthma (87), RA (50) | Calreticulin (CRT)  Cit-CRT (through PAD4)  Citrullineated proteins | ELISA | N/A |
| Perry 2017 | UK | 53/50 | Cases: adult non-CF bronchiectasis with co-existent RA (53)  Controls: RA without lung disease (50) | CCP  RF | ELISA (CCP)  Automated analyser (RF) | N/A |
| McDermott 2022 | USA | 57/360 | Cases: RA with bronchiectasis (57)  Controls: RA without lung disease (360) | CCP  RF | N/A | N/A |

**Bronchiectasis: manually added**

| **Study author & year** | **Primary country** | **Cases/ controls (n)** | **Severity of cases/details of controls (if relevant)** | **Antigens studied** | **Assay used** | **Functional assays** |
| --- | --- | --- | --- | --- | --- | --- |
| Skopelja-Gardner 2018 | USA | 58/0 | Cases: bronchiectasis patients from two cohorts (n=16, n=42 respectively) | BPI | ELISA | N/A |

**Asthma: Cytokeratins**

| **Study author & year** | **Primary country** | **Cases/ controls (n)** | **Severity of cases/details of controls (if relevant)** | **Antigens studied** | **Assay used** | **Functional assays** |
| --- | --- | --- | --- | --- | --- | --- |
| Nahm 2002 | South Korea | 50/54 | Cases: non-allergic asthma (23), allergic asthma (27)  Controls : non-smoker HC (34), SLE (20) | CK18 | Immunoblot | N/A |
| Mohammad 2016 | Egypt | 50/30 | Cases: allergic asthma (25), non-allergic asthma (25); of these mild (1), moderate (27), severe (22)  Controls: HC women (30) | CK18  TPO | ELISA (CK18),  N/A (TPO) | N/A |
| Ye 2006 | South Korea | 140/88 | Cases: aspirin intolerant (79), aspirin tolerant (61)  Controls: HC (88) | CK8  CK18  CK19  TTG (IgG, IgA)  C1q-binding immune complex (CIC)  ANA | ELISA (all)  Immunoblot (CK8, CK18, CK19 only) | N/A |

**Asthma: Routine autoantibodies in clinical practice**

| **Study author & year** | **Primary country** | **Cases/ controls (n)** | **Severity of cases/details of controls (if relevant)** | **Antigens studied** | **Assay used** | **Functional assays** |
| --- | --- | --- | --- | --- | --- | --- |
| Ye 2006 | See cytokeratins |  |  |  |  |  |
| Tamai 2017  Focuses on asthma | Japan | 202/0 | Cases: COPD (92), asthma (110) | ANA (≥1:160)  ANCA  RF  CCP  MPO  PR3 | IIF (ANA)  Immunoturbidometry (RF)  Chemiluminescent microparticle immuonassay (CCP)  Unclear (MPO, PR3) | N/A |
| Murkherjee 2018 | Canada | 51/15 | Cases: ICS dependent eosinophilic asthma (13), OCS dependent eosinophilic asthma (20), mixed phenotype eosinophilic asthma (18), neutrophilic infective asthma (13)  Controls: HC (15) | ANA/ENA  EPX  Both in sputum however they also tested ANA/ENA in serum | Line blot and IIF (ANA/ENA)  Immunoprecipitation (EPX)  Sputum | Eosinophili degranulation |
| Qin 2019 | China | 50/24 | Cases: all stable asthma; mild (15), moderate (18), severe (17)  Controls: HC (24) | PR3, P0, SSA, SSB, Scl70, Jo-1, U1-SnRNP, Sm, MPO, TPO | Multiplex bead array  Sputum/serum | N/A |

**Asthma: Miscellaneous**

| **Study author & year** | **Primary country** | **Cases/ controls (n)** | **Severity of cases/details of controls (if relevant)** | **Antigens studied** | **Assay used** | **Functional assays** |
| --- | --- | --- | --- | --- | --- | --- |
| Liu 2012 | USA | 99/60 | Cases: mild/intermittent (34), moderate persistent (49), severe (16)  Controls: HC (60) | Collagen V (IgG, A, M, E)  Protein array 9000 (6 pooled samples who were positive for ColV ab) | ELISA | N/A |
| Taille 2016 | France | 260/40 | Cases: severe (188), mild-mod (72)  Controls: HC (40) | Periplakin (IgG, IgE) | Western blot | N/A |
| Ji 2024 | China | 137/96 | Cases: mild-moderate (70), severe (67)  Controls: HC (96) | 11 cytokines, focused on IL-33 IgG, A, M | Luciferase immunoprecipitation system | IL-33 neutralisation assay with ELISA/qPCR and BALBc mice with rIL-33-induced lung inflammation |

**Sarcoidosis: Routine autoantibodies in clinical practice**

| **Study author & year** | **Primary country** | **Cases/ controls (n)** | **Severity of cases/details of controls (if relevant)** | **Antigens studied** | **Assay used** | **Functional assays** |
| --- | --- | --- | --- | --- | --- | --- |
| Kobak 2014 | Turkey | 42/90 | Cases: sarcoidosis (42)  Controls: RA (45), HC (45) | CCP IgG  RF IgM | ELISA (CCP)  Nephelometry (RF) | N/A |
| Starshinova 2022 | See below in vimentin |  |  |  |  |  |
| Shi 2022 | China | 154/100 | Cases: histologically proven sarcoidosis (154)  Controls: HC or assessed for arthralgia (100) | ANA (≥1:100)  RF  ENA  CCP  dsDNA  β2GP1  Cardiolipin  TPO  Thyroglobulin  ANCA | IIF (ANA)  Immunoblot (ENA)  ELISA (CCP, β2GP1, cardiolipin, TPO, thyroglobulin, ANCA) | N/A |

**Sarcoidosis: Vimentin**

| **Study author & year** | **Primary country** | **Cases/ controls (n)** | **Severity of cases/details of controls (if relevant)** | **Antigens studied** | **Assay used** | **Functional assays** |
| --- | --- | --- | --- | --- | --- | --- |
| Malkova 2021 | Russia | 93/68 | Cases: sarcoidosis (93)  Controls: HC (40), TB (28) | Vimentin | ELISA | N/A |
| Starshinova 2022  Same cohort as Malkova | Russia | 93/95 | Cases: histologically verified sarcoidosis (93)  Controls: non-infectious lung disease (COPD 25, GPA 15, alveolitis 15), HC/TB contacts (40) | Mutated citrullinated vimentin (MCV), then CCP and citrullinated vimentin (Sa) and non-modified vimentin | ELISA | N/A |

**Sarcoidosis: Miscellaneous**

| **Study author & year** | **Primary country** | **Cases/ controls (n)** | **Severity of cases/details of controls (if relevant)** | **Antigens studied** | **Assay used** | **Functional assays** |
| --- | --- | --- | --- | --- | --- | --- |
| Khassawneh 2022 | USA | 236/100 | Cases: sarcoidosis with only pulmonary involvement (POS, 106), sarcoidosis with extrapulmonary involvement (EPS, 120)  Controls: normal controls, NC (101) | Multiple/unbiased (IgG/M) | Protein array (128 antigens/internal controls) | N/A |

**Sarcoidosis: Unbiased**

| **Study author & year** | **Primary country** | **Cases/ controls (n)** | **Severity of cases/details of controls (if relevant)** | **Antigens studied** | **Assay used** | **Functional assays** |
| --- | --- | --- | --- | --- | --- | --- |
| Haggmark 2015 | Sweden | 279/66 | BAL samples: Initial screen with 40/33, then remaining 272 samples added for verification.  Cases: sarcoidosis with Lofgren’s (140), sarcoidosis without Lofgren’s (139)  Controls: HC (49), mild allergic asthma (17)  Serum samples: sarcoidoisis (123), HC (18) | Multiple/unbiased (included ZNF688, ARFGAP1, NCOA2, MRPL43) | Protein microarray (3072 antigens)  Suspension bead array  Immunohistochemistry on human tisuse | N/A |
| Baerlecken 2020 | Germany | 82/259 | Derivation cohort  Cases: sarcoidosis (24)  Controls: SLE (36), febrile infectious disease (46), PET controls (64), HC (100)  Second validation cohort  Cases: sarcoidosis (58)  Controls: HC (13) | Multiple/unbiased (focused on NELF-E) | Planar protein array using E. coli host system with 28,000 full and partial recombinant proteins of fetal human brain cDNA  ELISA (negative elongation factor E, NELF-E) | N/A |
| Peng 2024 | USA | 186/200 | Cases: sarcoidosis (186)  Controls: HC (100), pulmonary TB (70), lung cancer (30) | Previously unbiased with PhIP-Seq, now focused: chain A, cofilin μ | ELISA | N/A |

**Other: Autoimmune pulmonary alveolar proteinosis**

| **Study author & year** | **Primary country** | **Cases/ controls (n)** | **Severity of cases/details of controls (if relevant)** | **Antigens studied** | **Assay used** | **Functional assays** |
| --- | --- | --- | --- | --- | --- | --- |
| Nishimura 2017 | Japan | 223/50 | Cases: mixed with IIP (52), sarcoidosis (50), aPAP (50), pneumoconiosis (75)  Controls: HC (50) | GM-CSF | ELISA | N/A |
| Katayama 2020 | Japan | 137/141 | Cases: sarcoidosis (92), hypersensitivity pneumonitis (45)  Controls: aPAP (110), age/sex matched HC (31) | GM-CSF | ELISA | N/A |
